# Supplementary material for: Machine Learning Approach for Determining the Formation of β-Lactam Antibiotic Complexes with Cyclodextrins Using Multispectral Analysis
Source: Molecules. 2019 Feb 19;24(4):743. doi: 10.3390/molecules24040743 (PMC6413071; doi:10.3390/molecules24040743)
Supplement: Supplementary file 1 [file molecules-24-00743-s001.pdf]

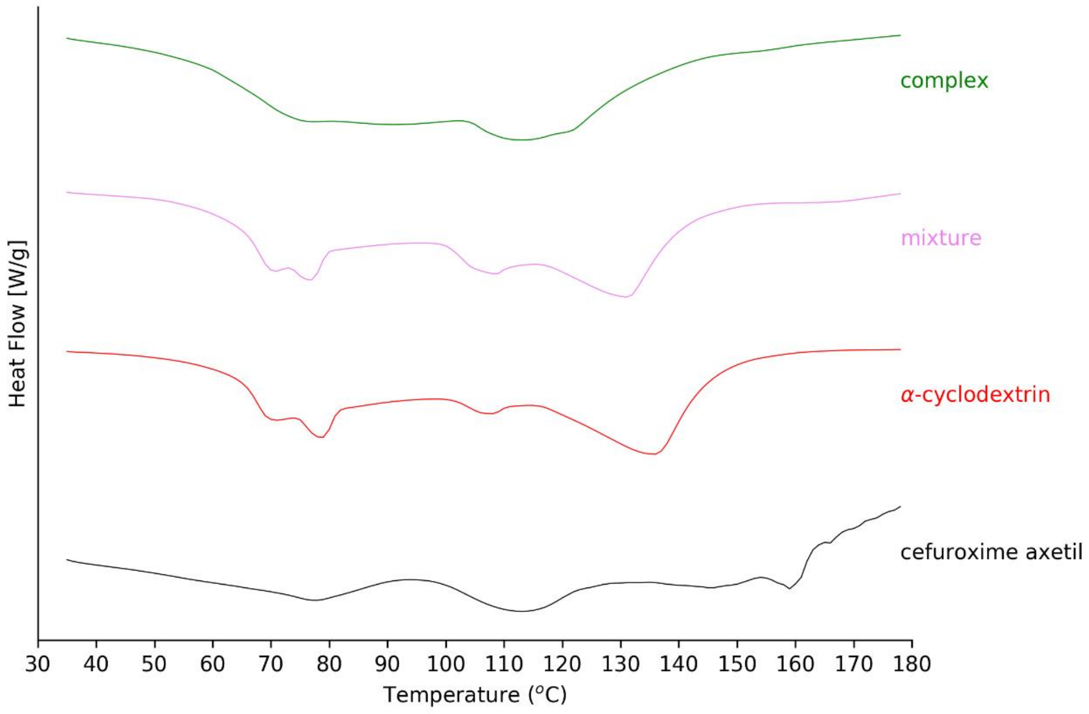

Figure S1. A DSC thermogram of CA – αCD system and its constituents.

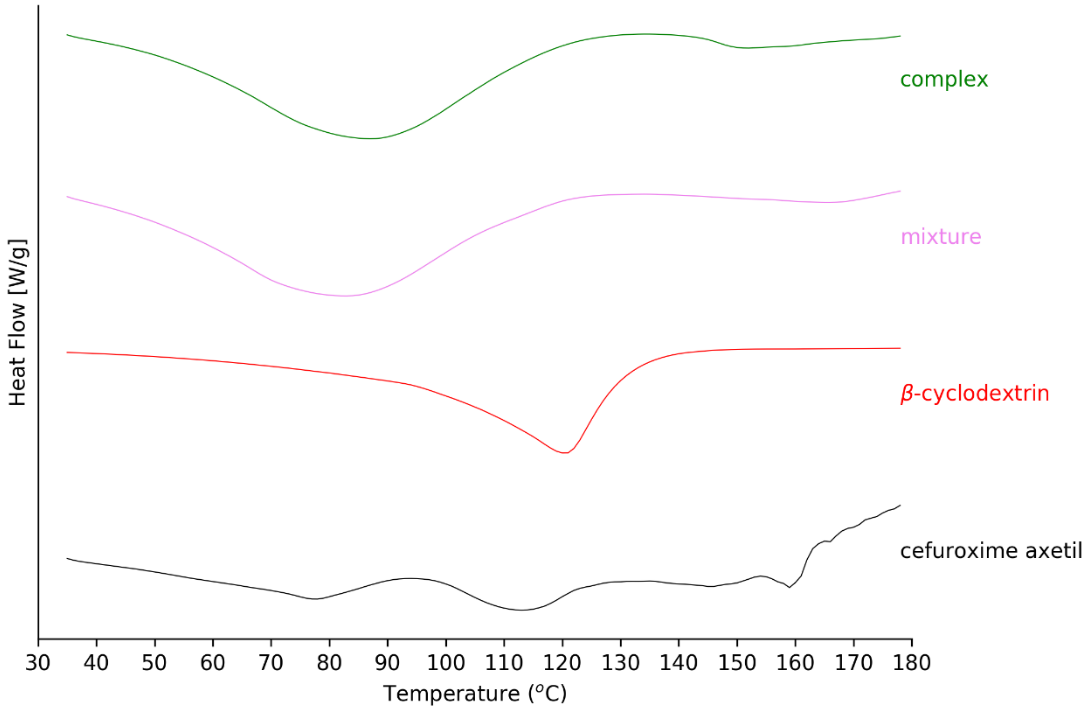

Figure S2. A DSC thermogram of CA – βCD system and its constituents.

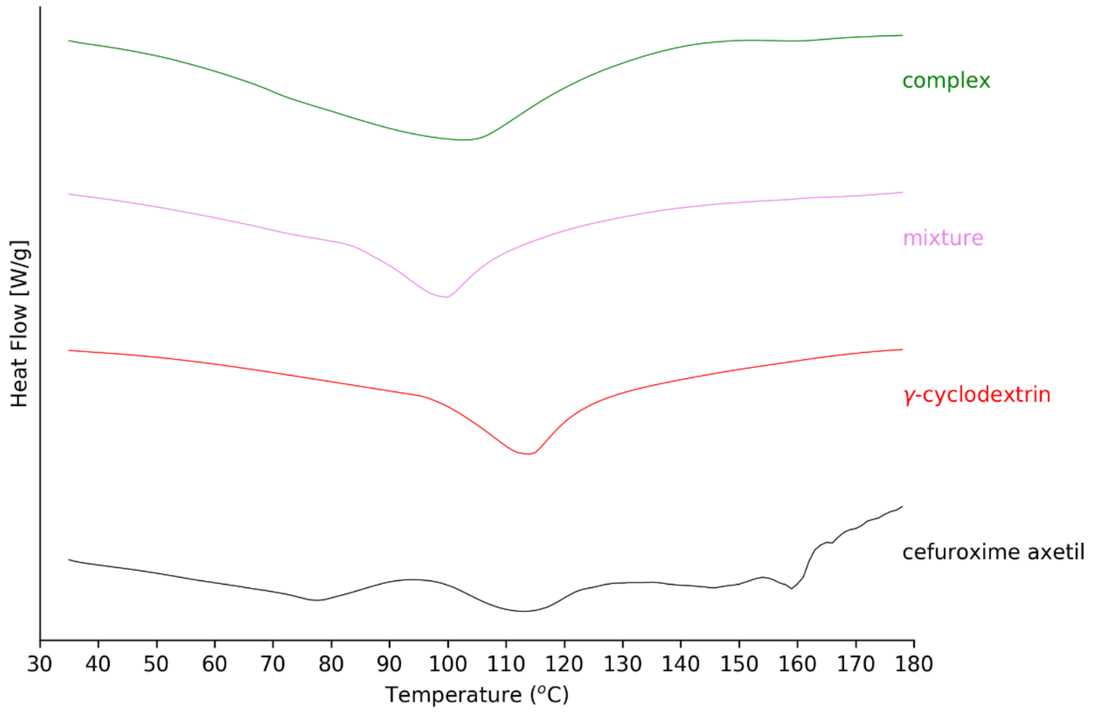

Figure S3. A DSC thermogram of CA – γCD system and its constituents.

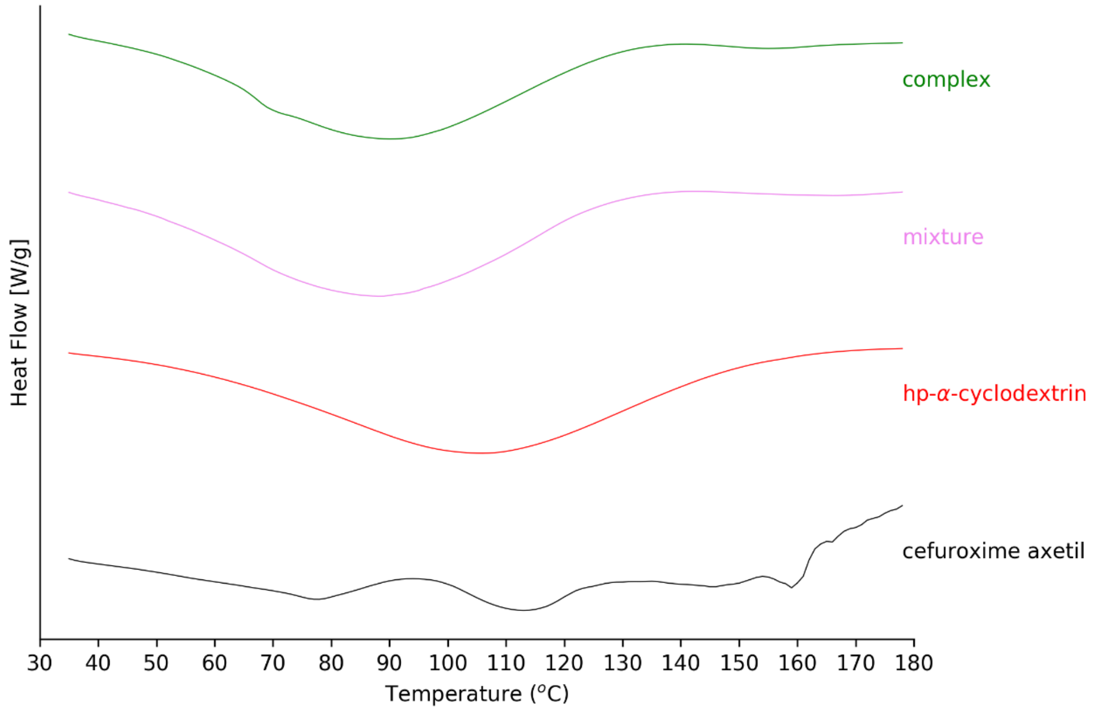

Figure S4. A DSC thermogram of CA – HPαCD system and its constituents.

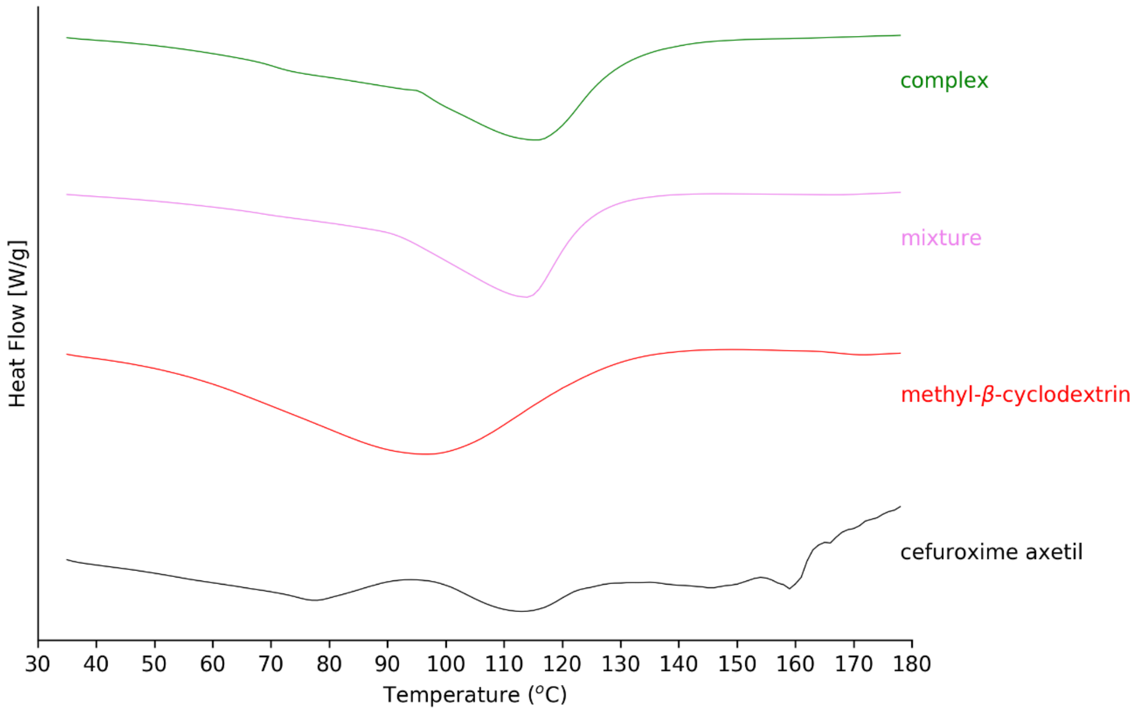

Figure S5. A DSC thermogram of CA – MβCD system and its constituents.

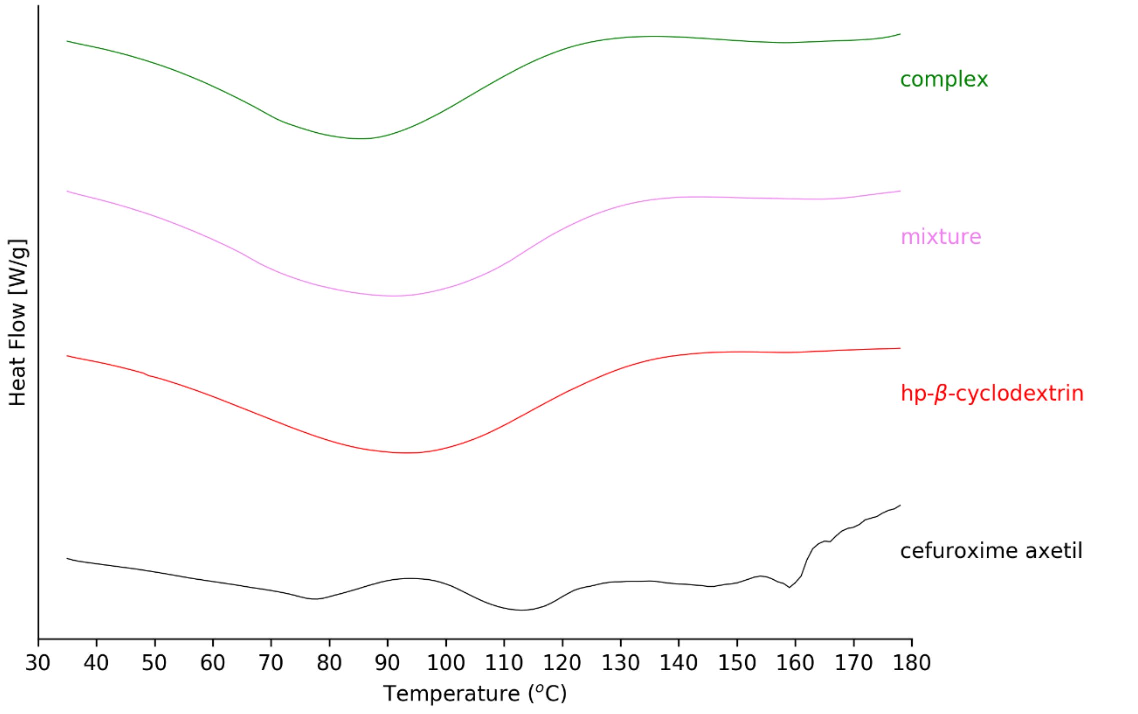

Figure S6. A DSC thermogram of CA – HPβCD system and its constituents.

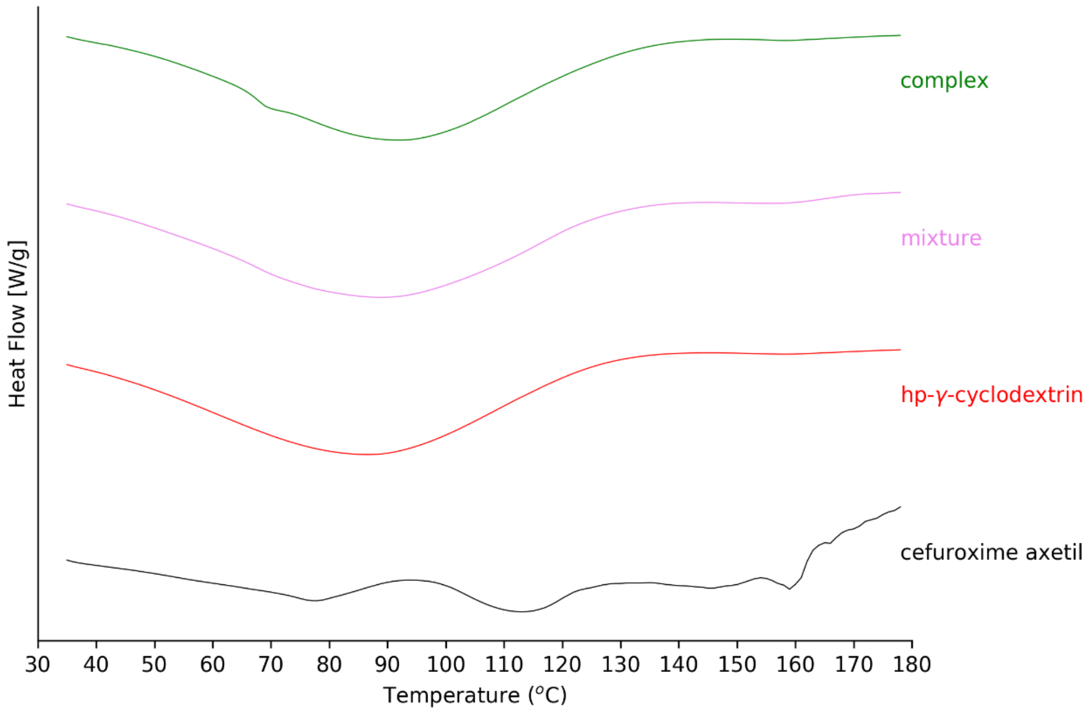

Figure S7. A DSC thermogram of CA – HPγCD system and its constituents.

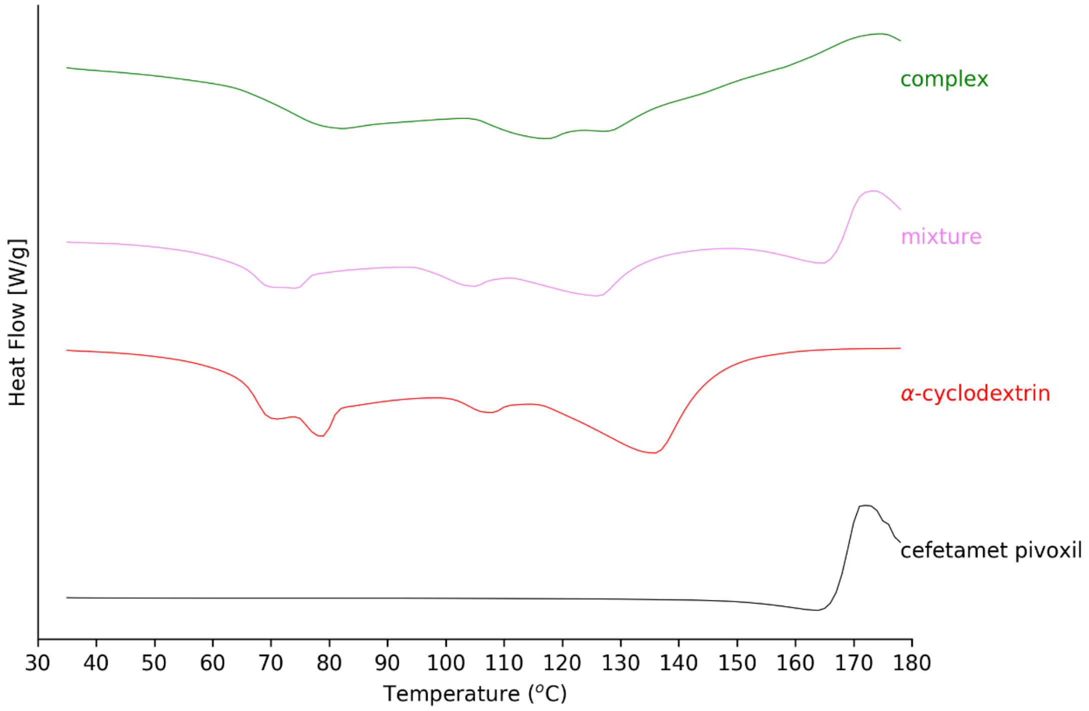

Figure S8. A DSC thermogram of CT – αCD system and its constituents.

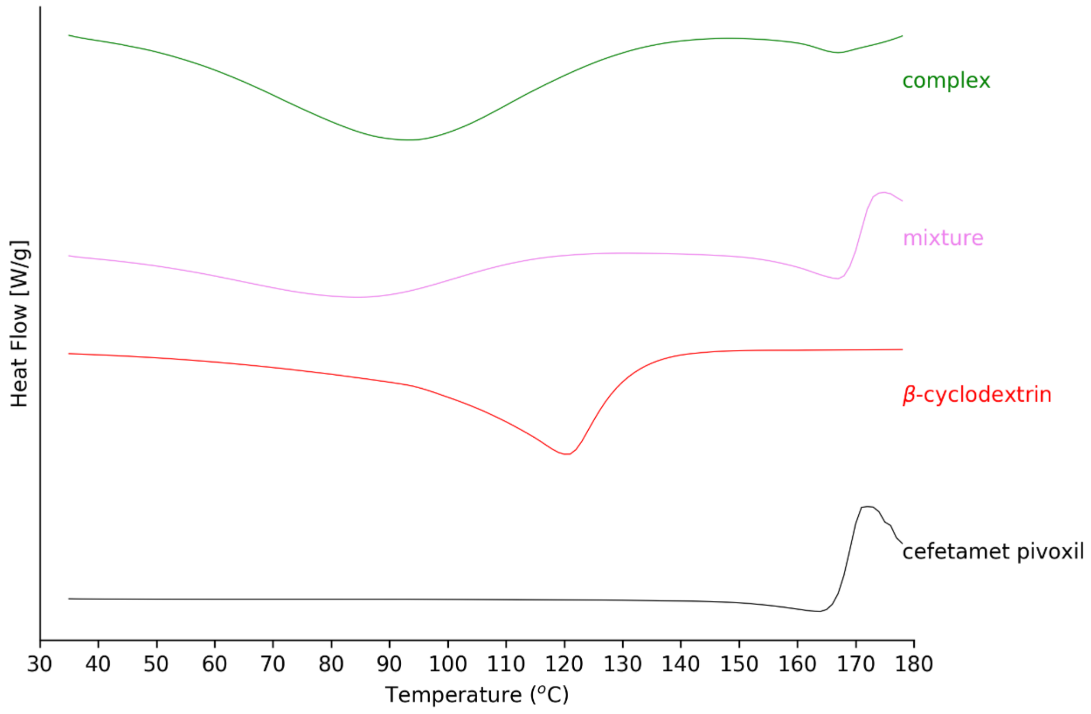

Figure S9. A DSC thermogram of CT – βCD system and its constituents.

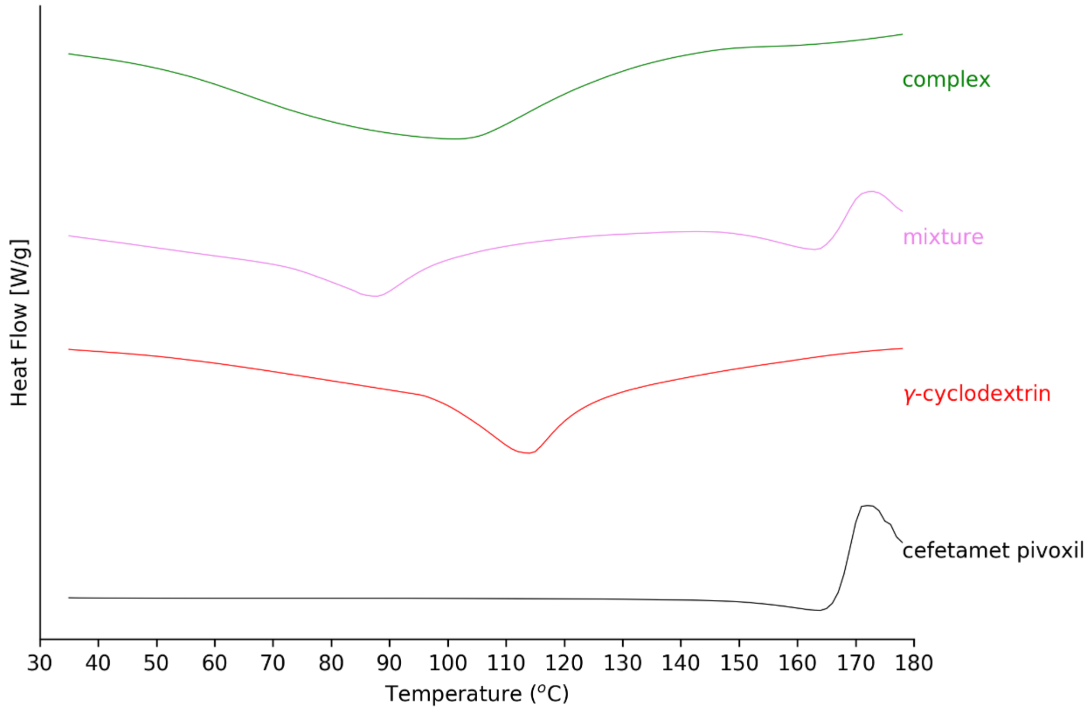

Figure S10. A DSC thermogram of CT – γCD system and its constituents.

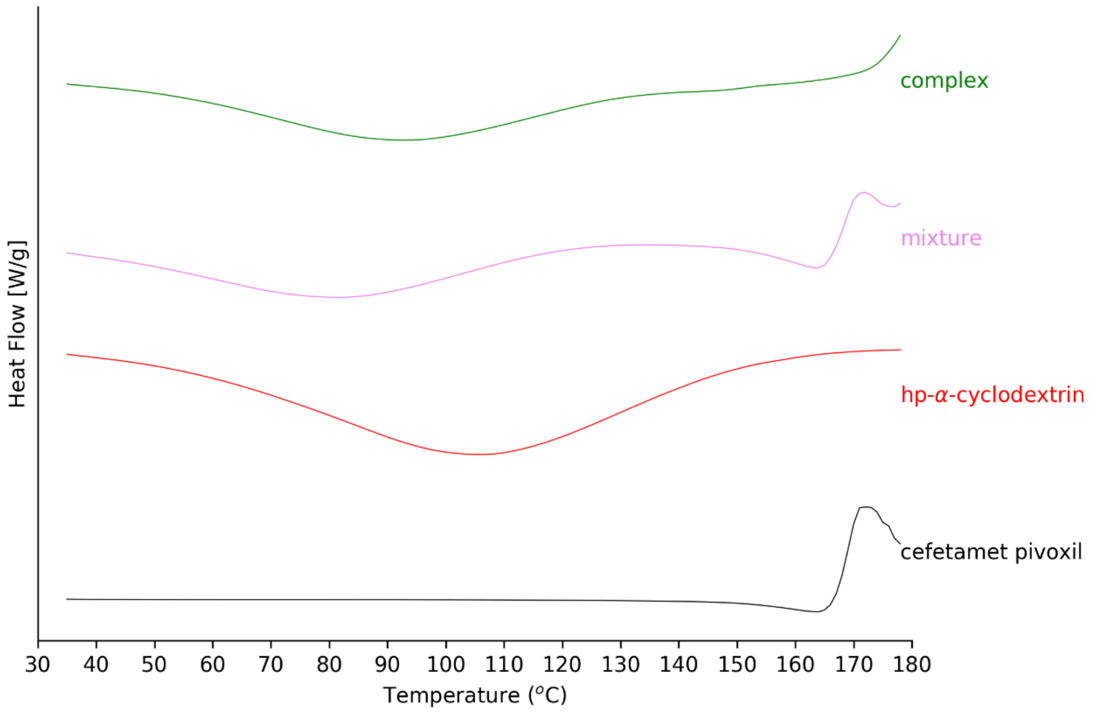

Figure S11. A DSC thermogram of CT – HPαCD system and its constituents.

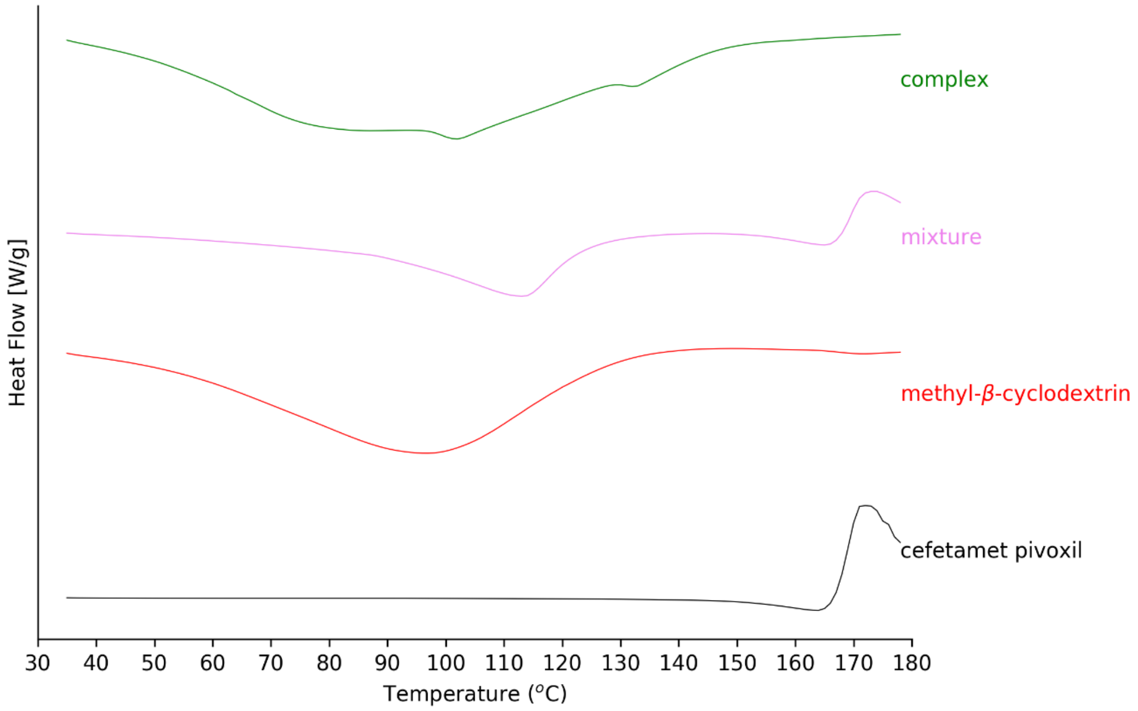

Figure S12. A DSC thermogram of CT – MβCD system and its constituents.

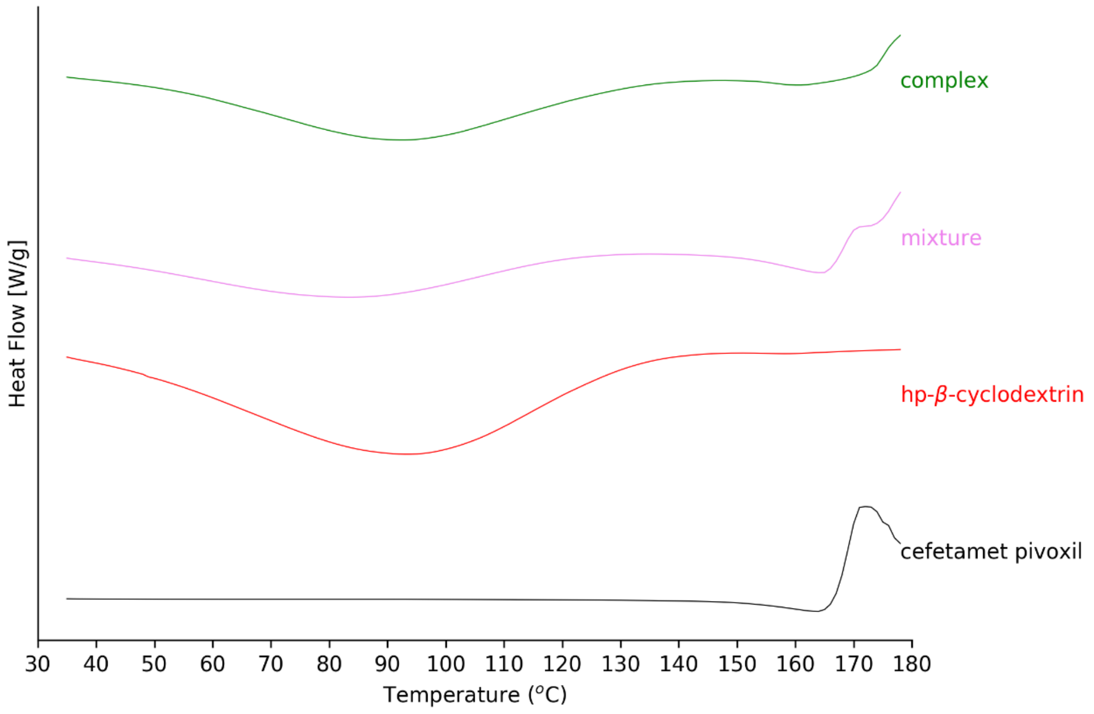

Figure S13. A DSC thermogram of CT – HPβCD system and its constituents.

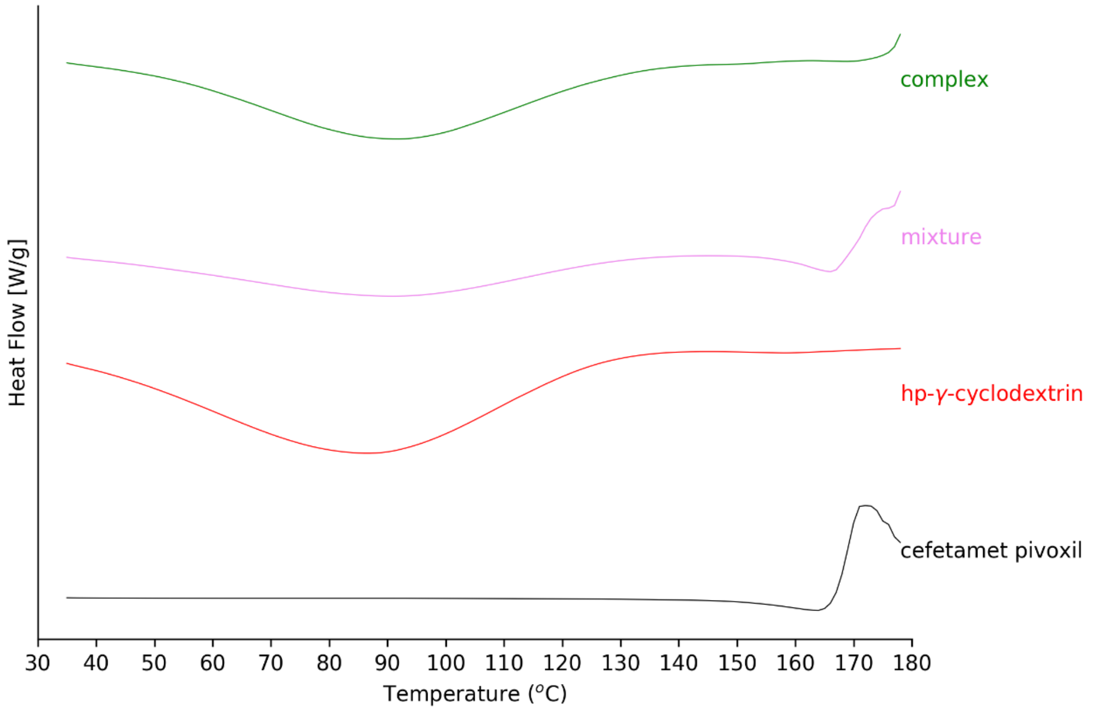

Figure S14. A DSC thermogram of CT – HPγCD system and its constituents.

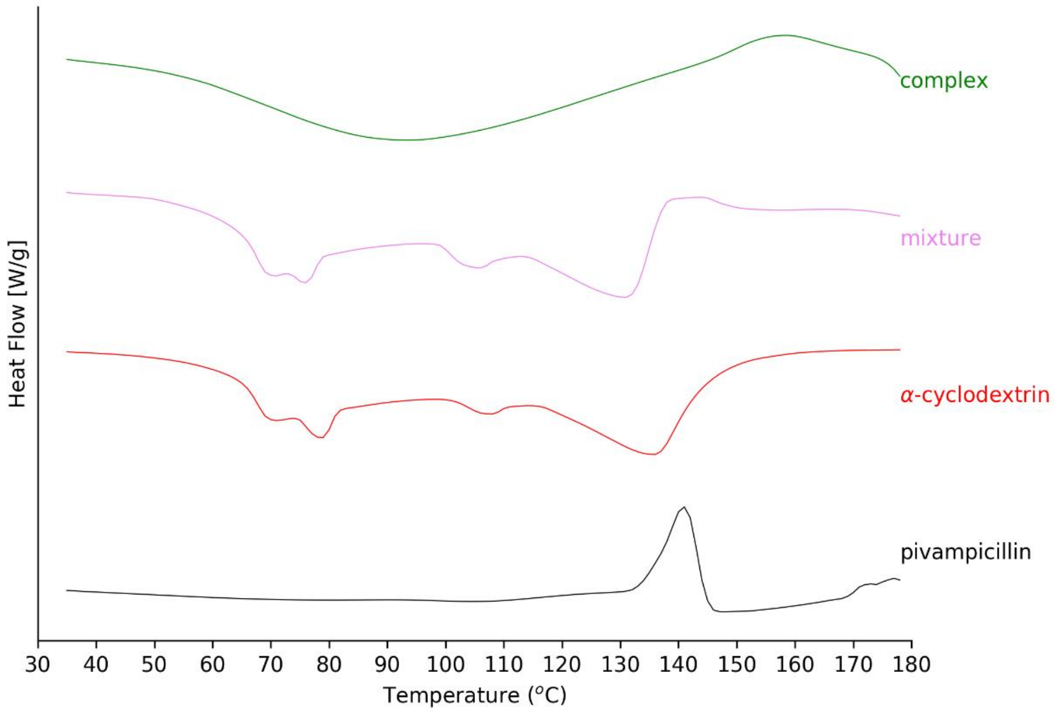

Figure S15. A DSC thermogram of PA – αCD system and its constituents.

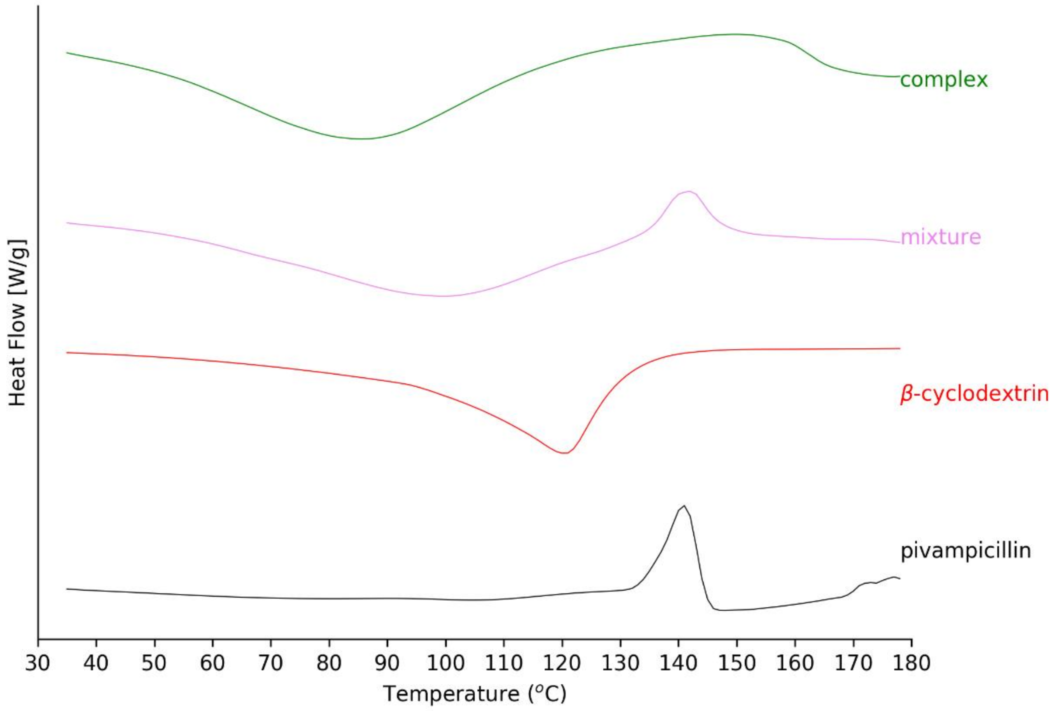

Figure S16. A DSC thermogram of PA – βCD system and its constituents.

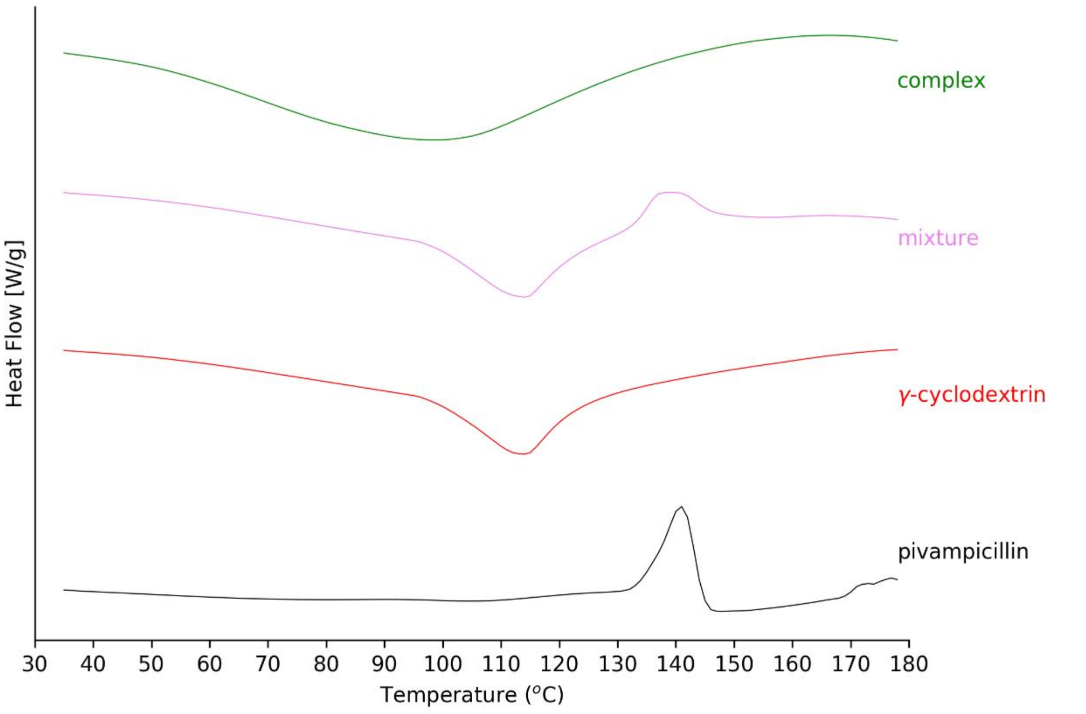

Figure S17. A DSC thermogram of PA – γCD system and its constituents.

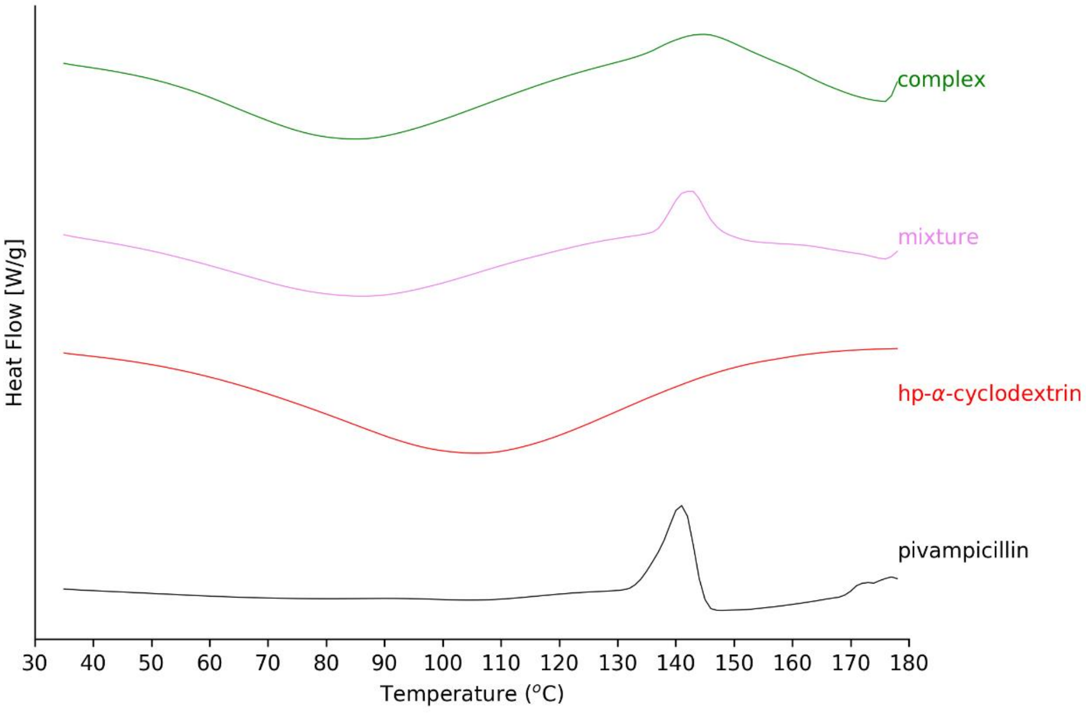

Figure S18. A DSC thermogram of PA – HPαCD system and its constituents.

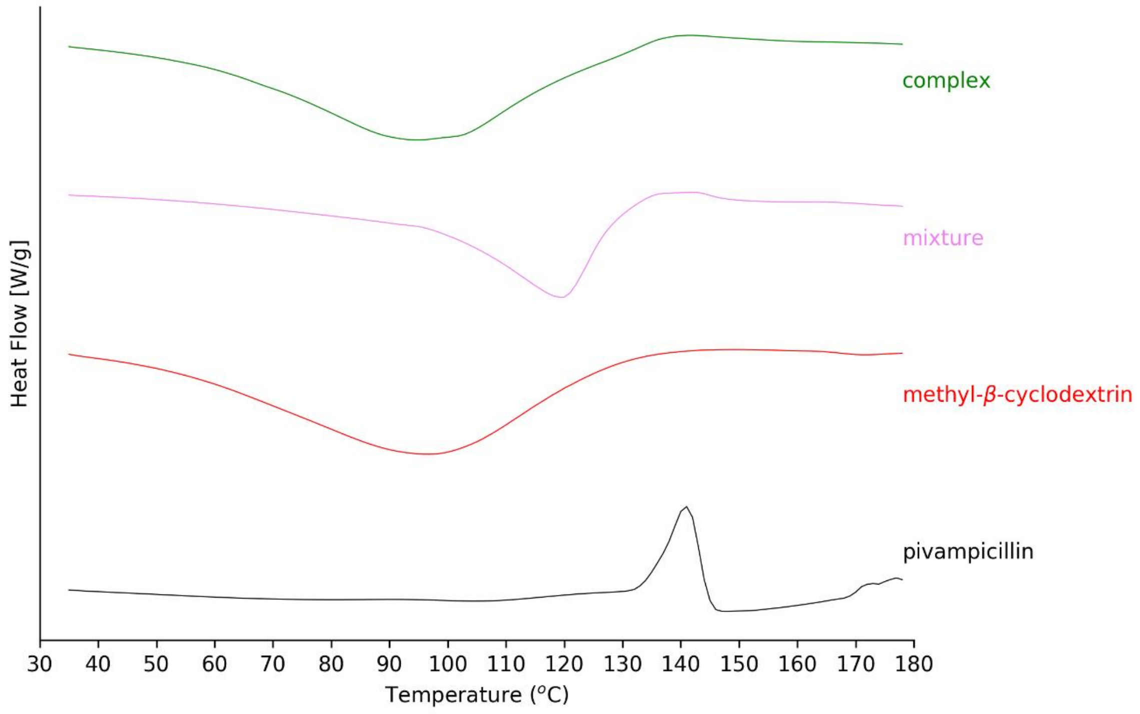

Figure S19. A DSC thermogram of PA – MβCD system and its constituents.

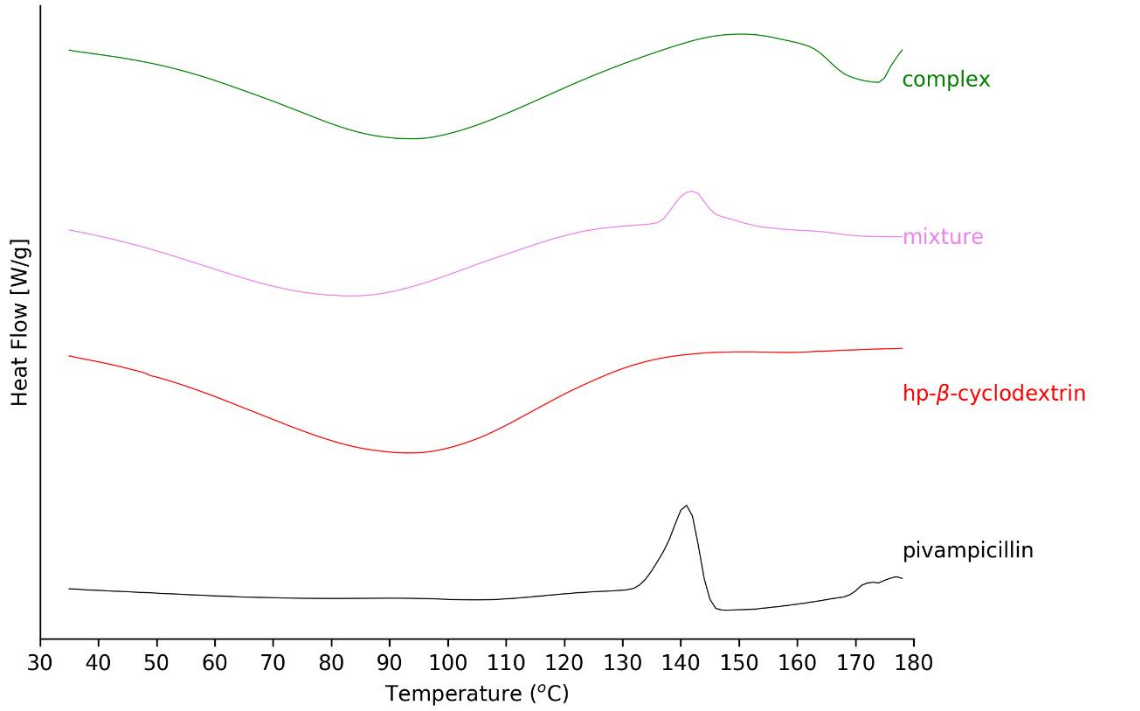

Figure S20. A DSC thermogram of PA – HPβCD system and its constituents.

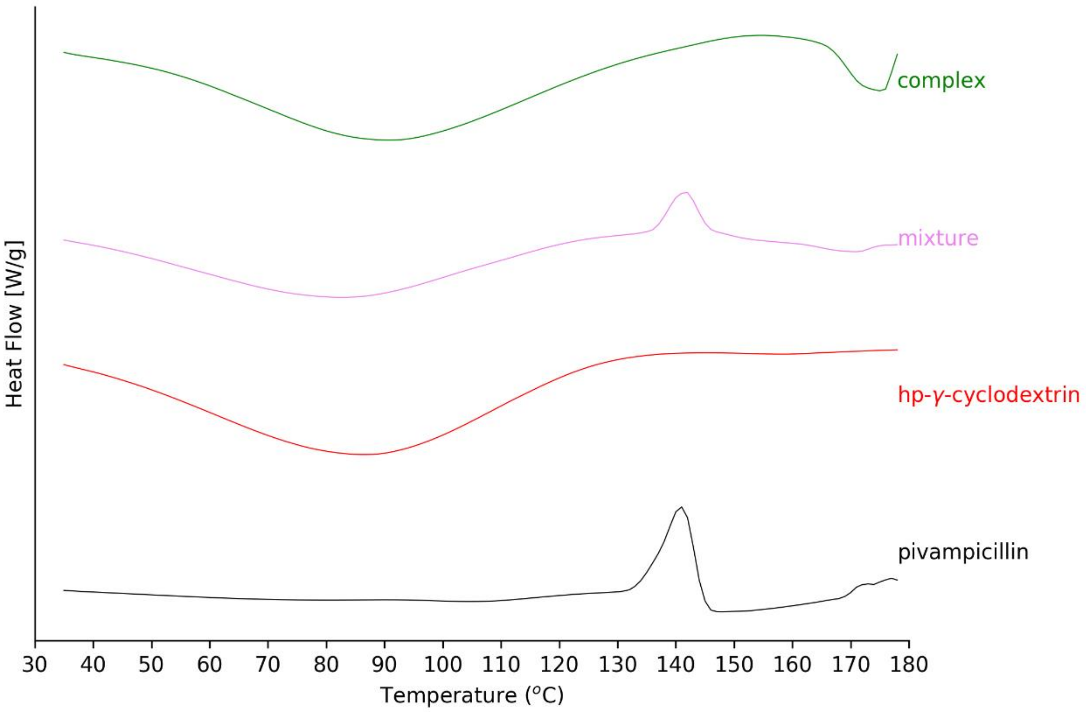

Figure S21. A DSC thermogram of PA – HPγCD system and its constituents.

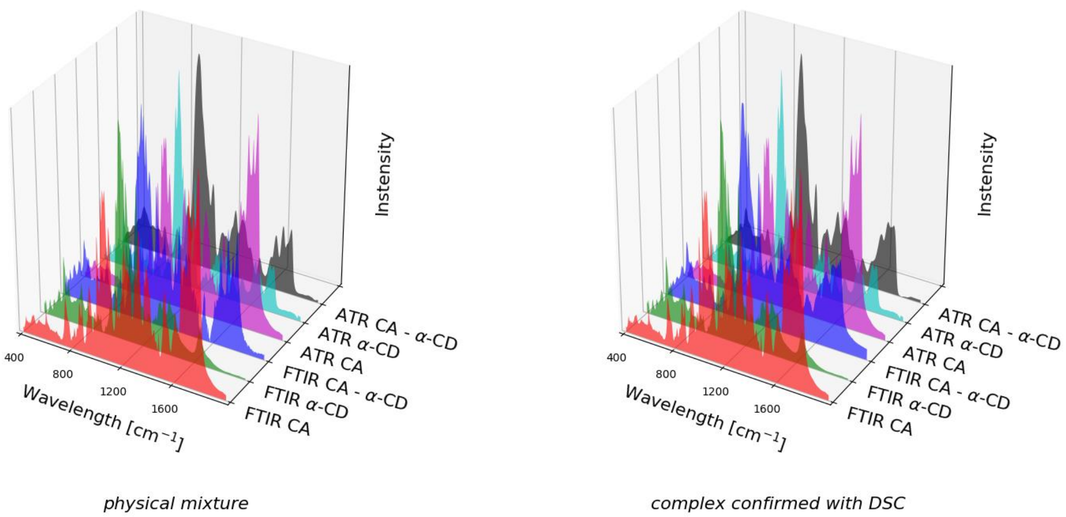

Figure S22. A records of input dataset containing features from FTIR and ATR spectra of CA – αCD systems and its constituents.

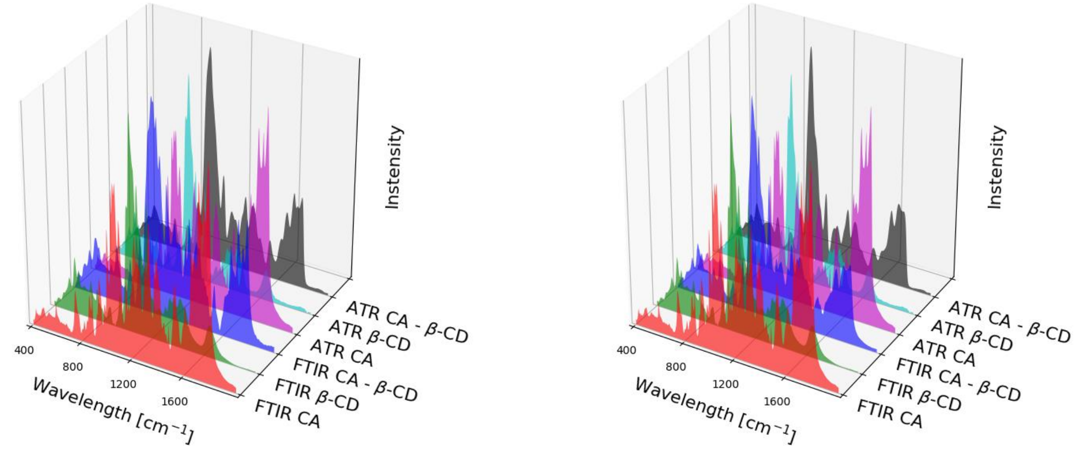

physical mixture *complex confirmed with DSC*

Figure S23. A records of input dataset containing features from FTIR and ATR spectra of CA – βCD systems and its constituents.

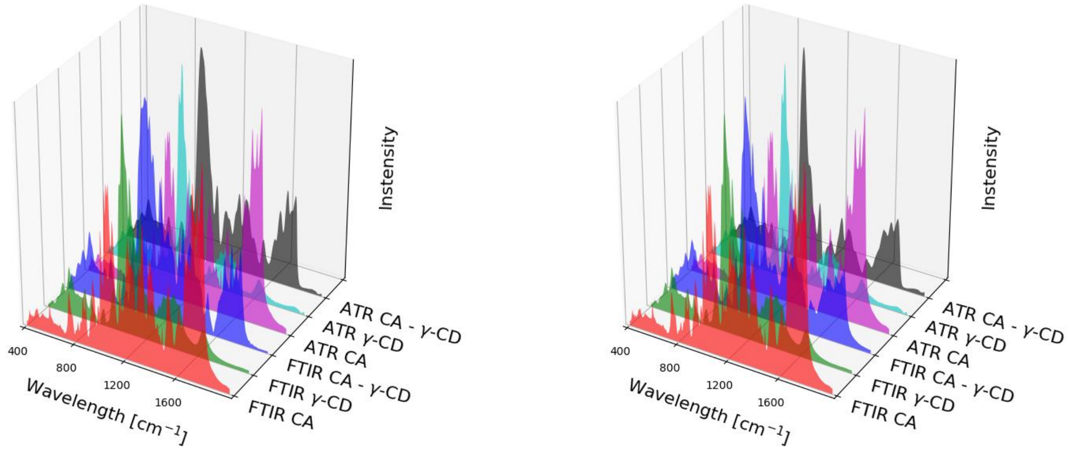

physical mixture *complex confirmed with DSC*

Figure S24. A records of input dataset containing features from FTIR and ATR spectra of CA – γCD systems and its constituents.

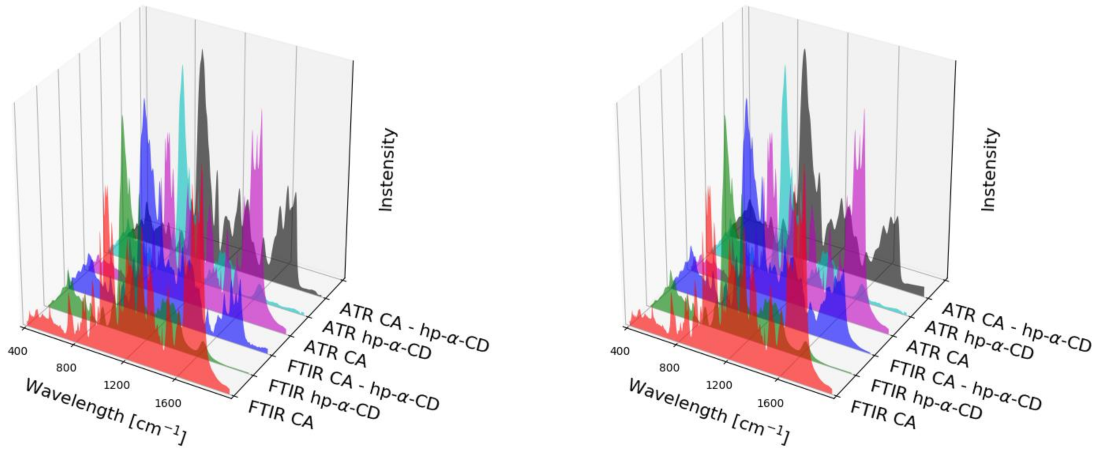

physical mixture *complex **not** confirmed with DSC*

Figure S25. A records of input dataset containing features from FTIR and ATR spectra of CA – HPαCD systems and its constituents.

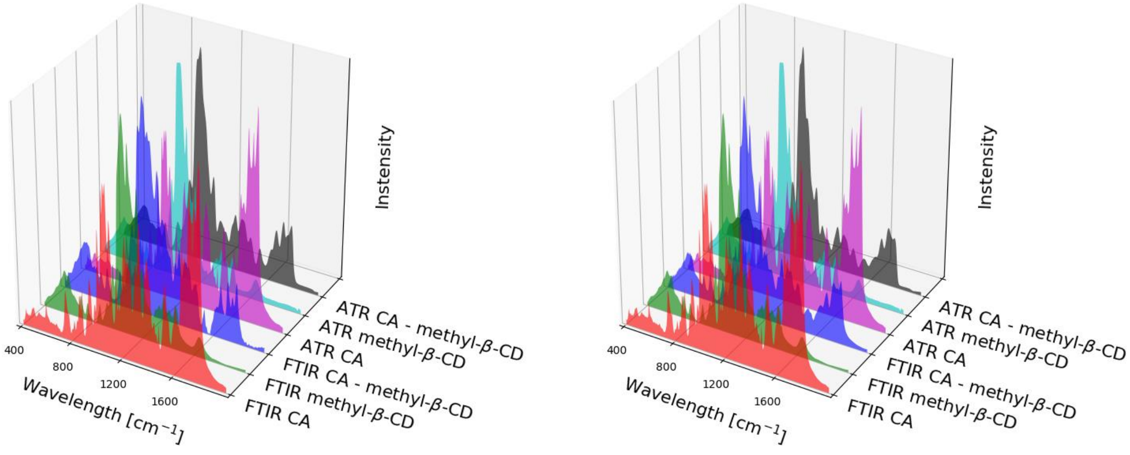

physical mixture *complex confirmed with DSC*

Figure S26. A records of input dataset containing features from FTIR and ATR spectra of CA – MβCD systems and its constituents.

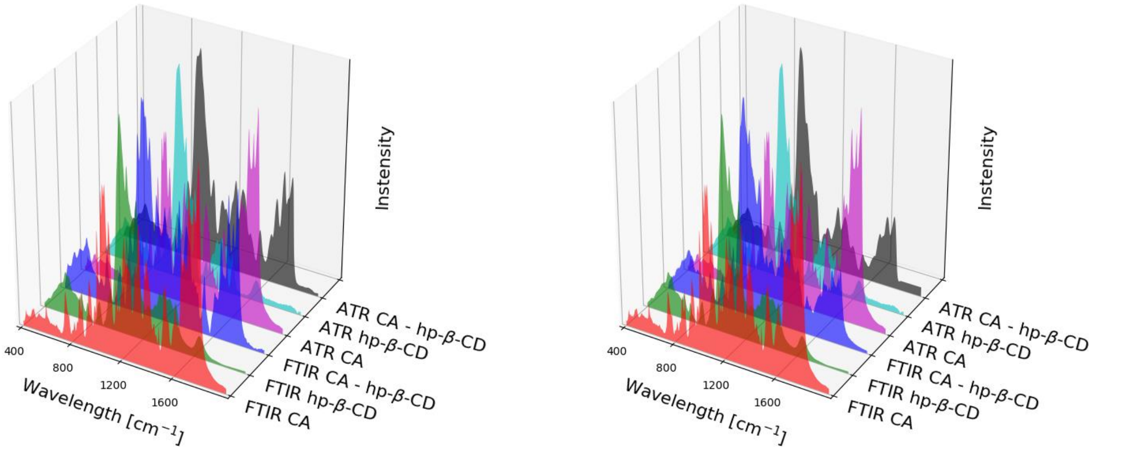

physical mixture *complex confirmed with DSC*

Figure S27. A records of input dataset containing features from FTIR and ATR spectra of CA – HPβCD systems and its constituents.

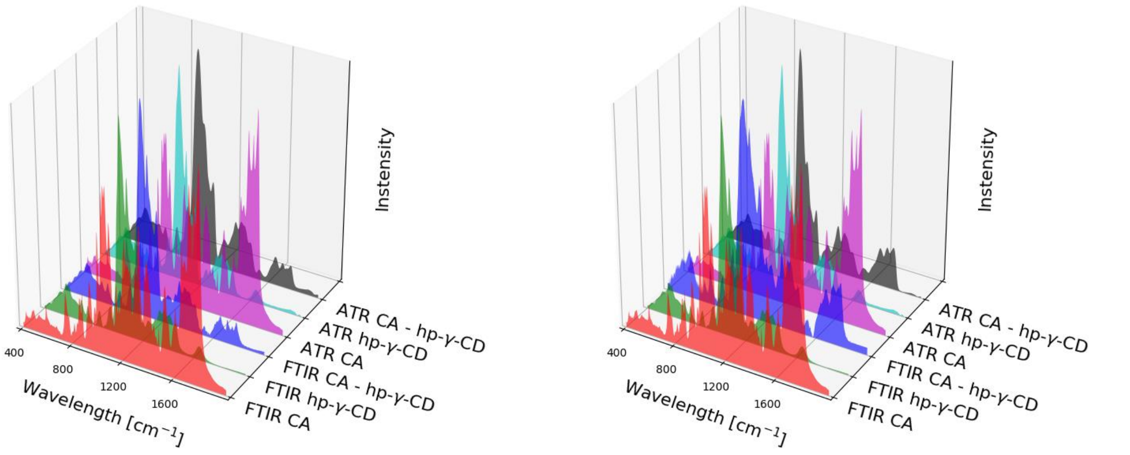

physical mixture *complex confirmed with DSC*

Figure S28. A records of input dataset containing features from FTIR and ATR spectra of CA – HPγCD systems and its constituents.

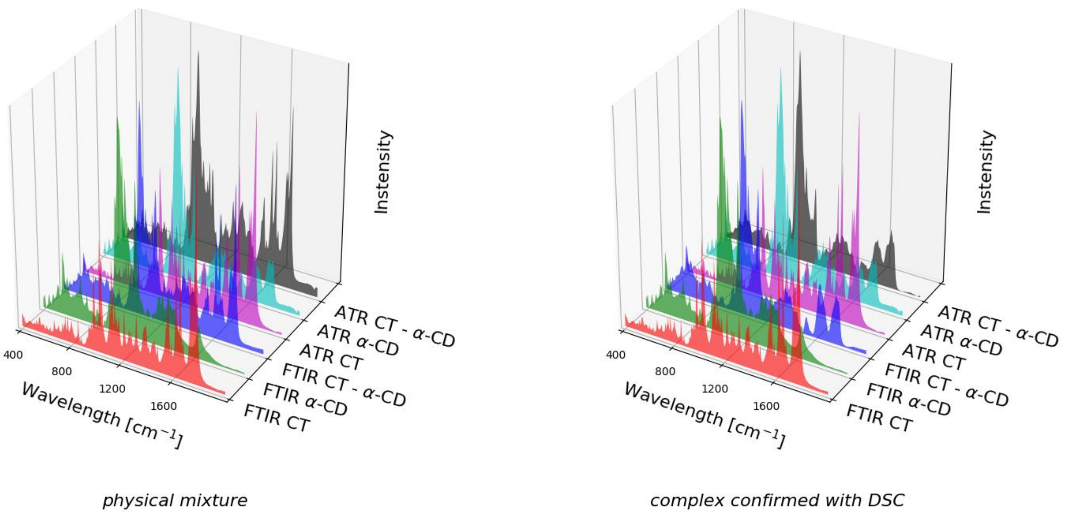

Figure S29. A records of input dataset containing features from FTIR and ATR spectra of CT – αCD systems and its constituents.

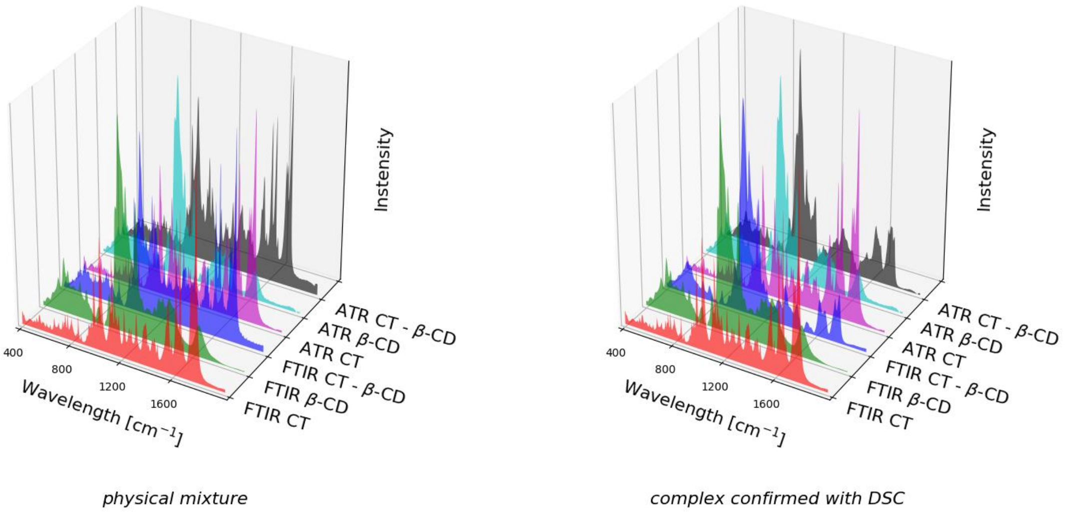

Figure S30. A records of input dataset containing features from FTIR and ATR spectra of CT – βCD systems and its constituents.

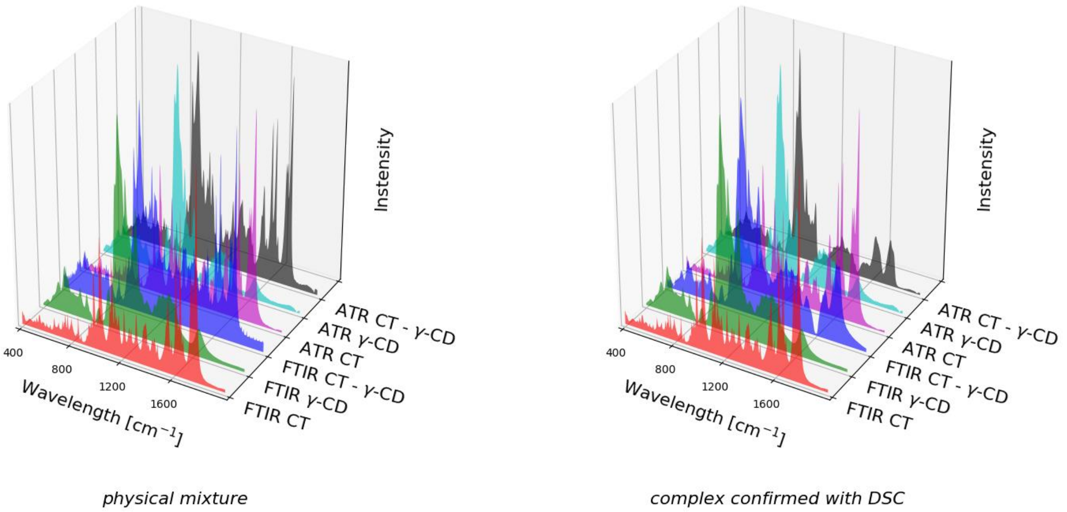

Figure S31. A records of input dataset containing features from FTIR and ATR spectra of CT – γCD systems and its constituents.

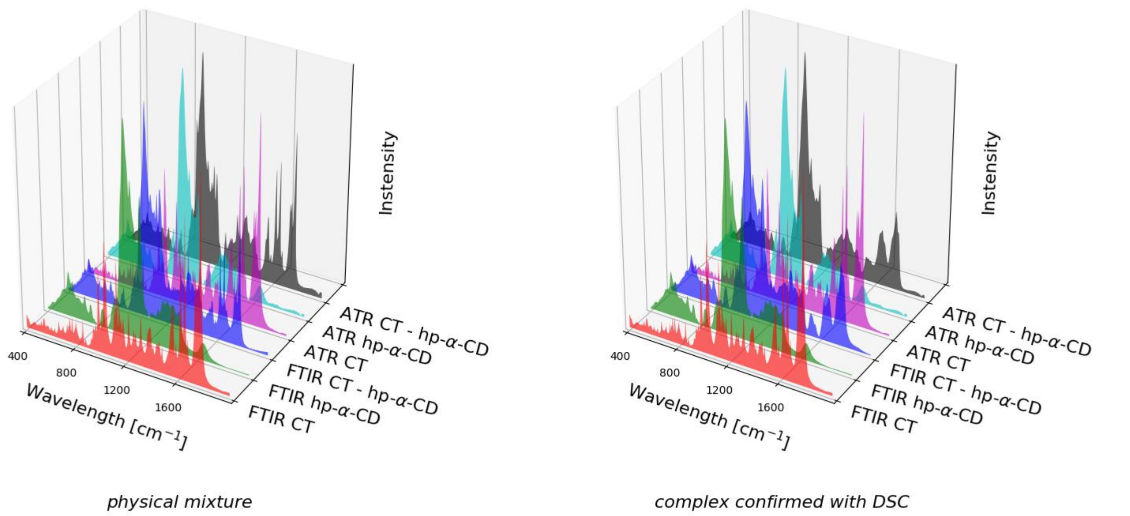

Figure S32. A records of input dataset containing features from FTIR and ATR spectra of CT – HPαCD systems and its constituents.

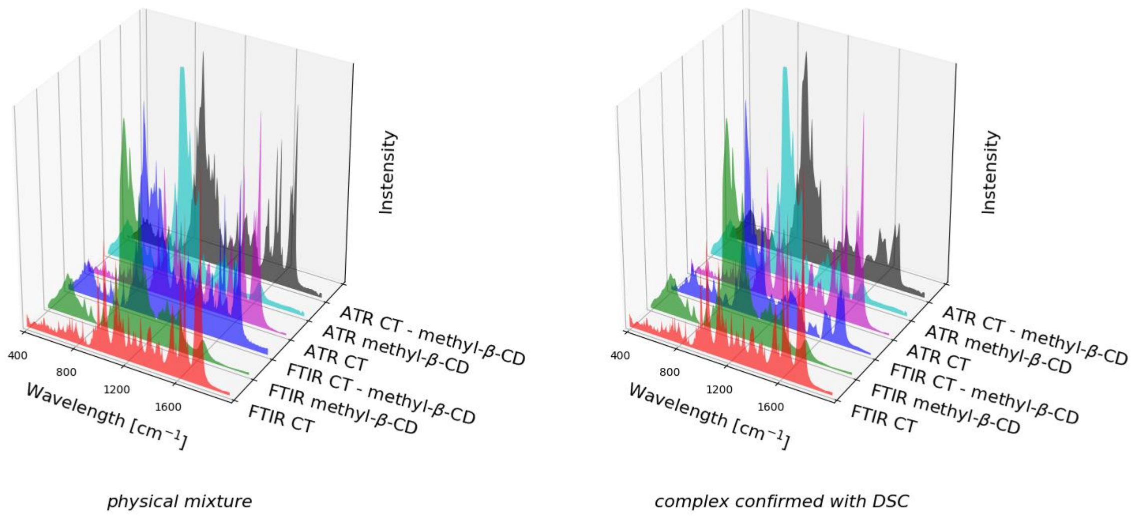

Figure S33. A records of input dataset containing features from FTIR and ATR spectra of CT – MβCD systems and its constituents.

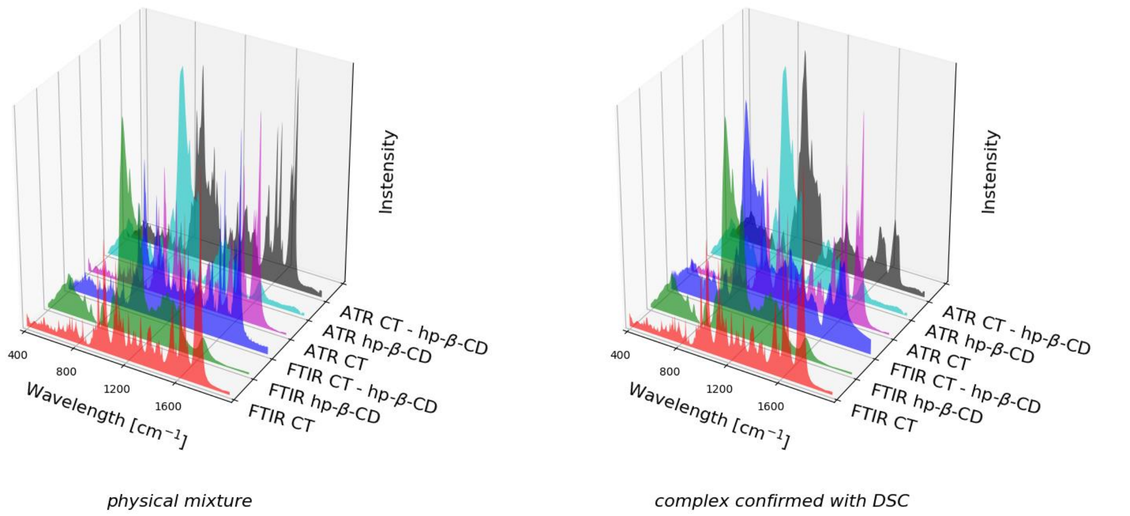

Figure S34. A records of input dataset containing features from FTIR and ATR spectra of CT – hpβCD systems and its constituents.

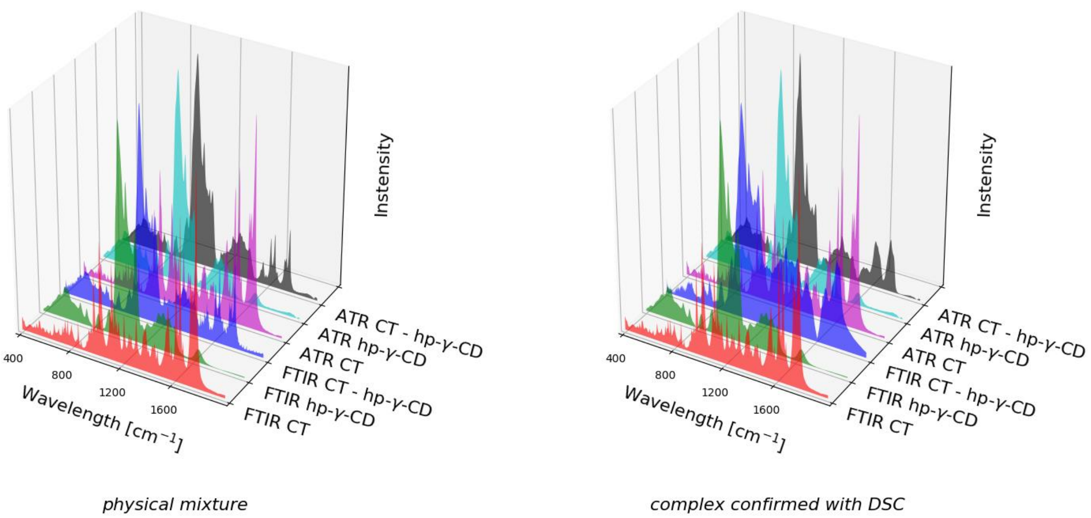

Figure S35. A records of input dataset containing features from FTIR and ATR spectra of CT – HPγCD systems and its constituents.

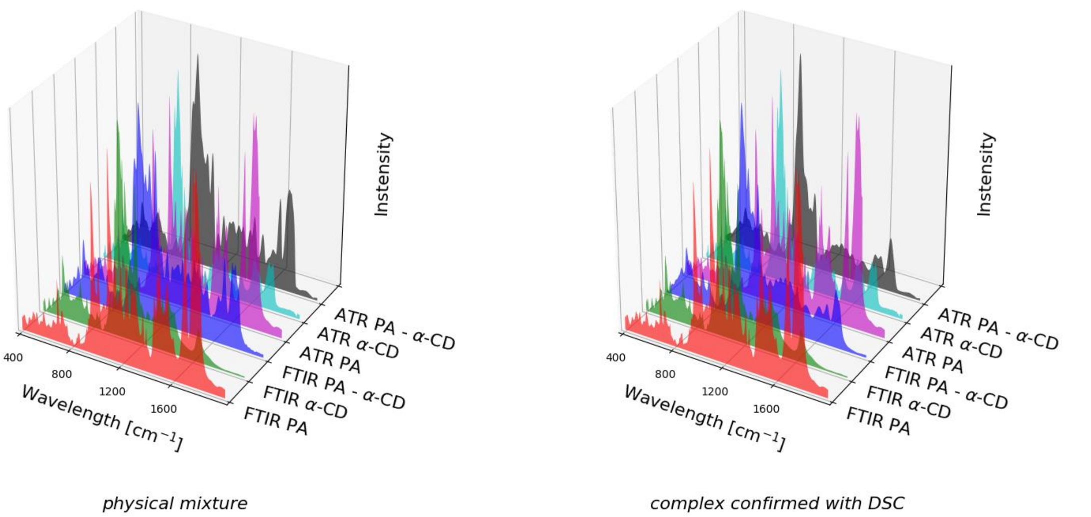

Figure S36. A records of input dataset containing features from FTIR and ATR spectra of PA – αCD systems and its constituents.

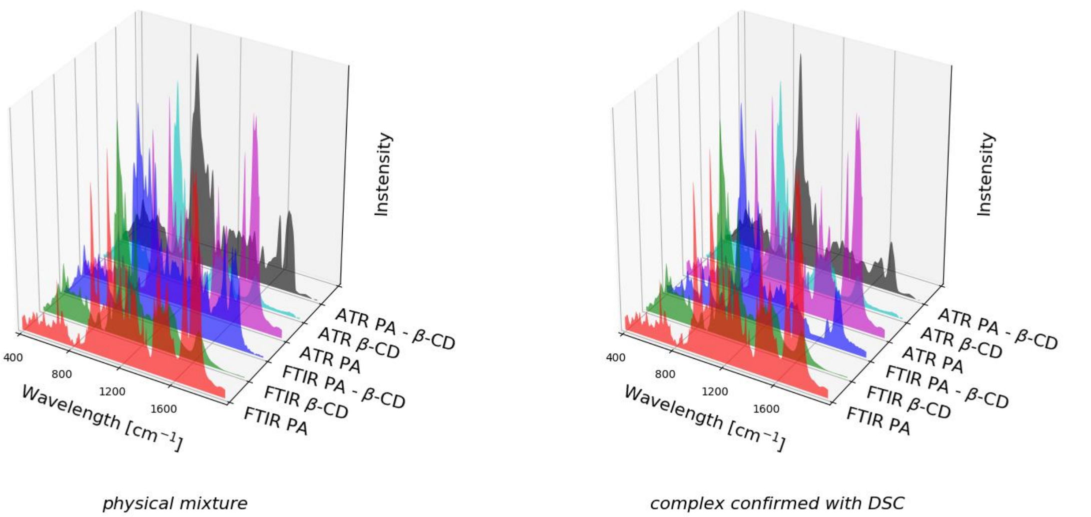

Figure S37. A records of input dataset containing features from FTIR and ATR spectra of PA – βCD systems and its constituents.

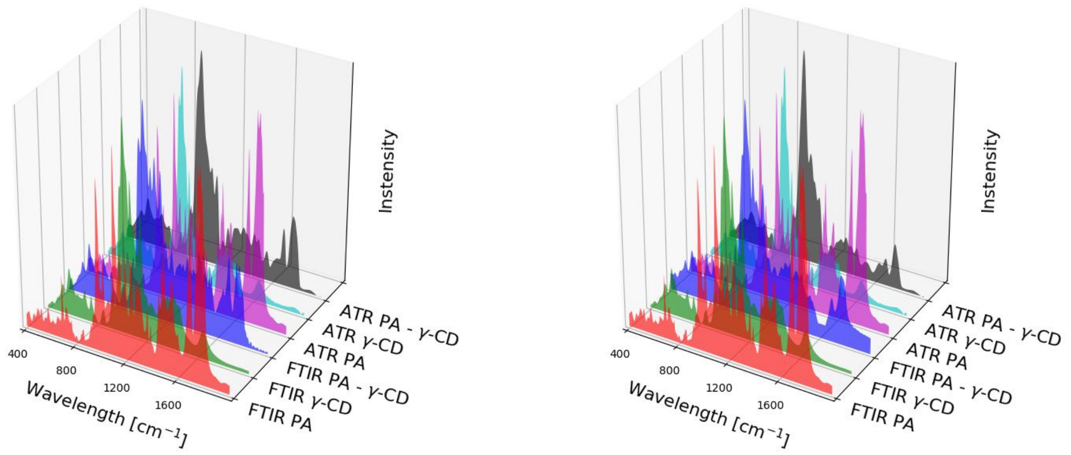

physical mixture *complex confirmed with DSC*

Figure S38. A records of input dataset containing features from FTIR and ATR spectra of PA – γCD systems and its constituents.

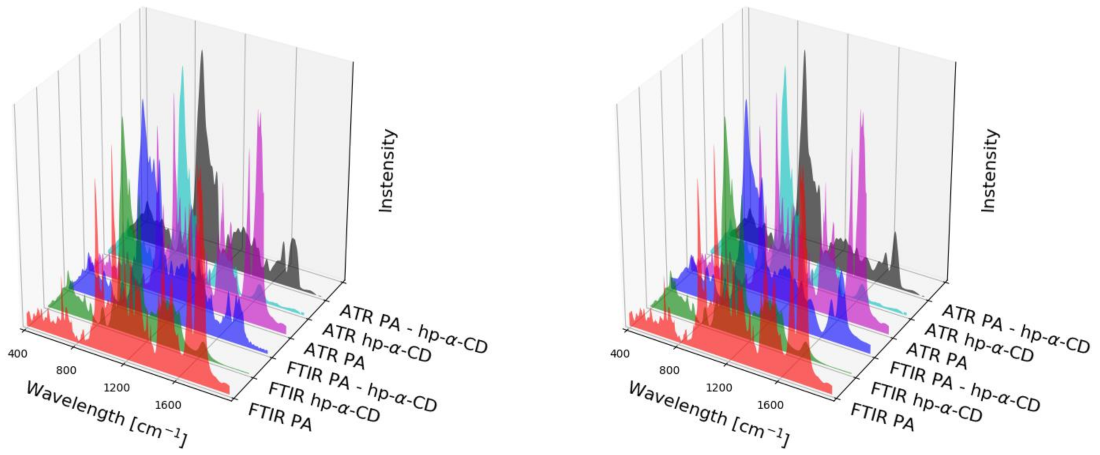

physical mixture *complex confirmed with DSC*

Figure S39. A records of input dataset containing features from FTIR and ATR spectra of PA – HPαCD systems and its constituents.

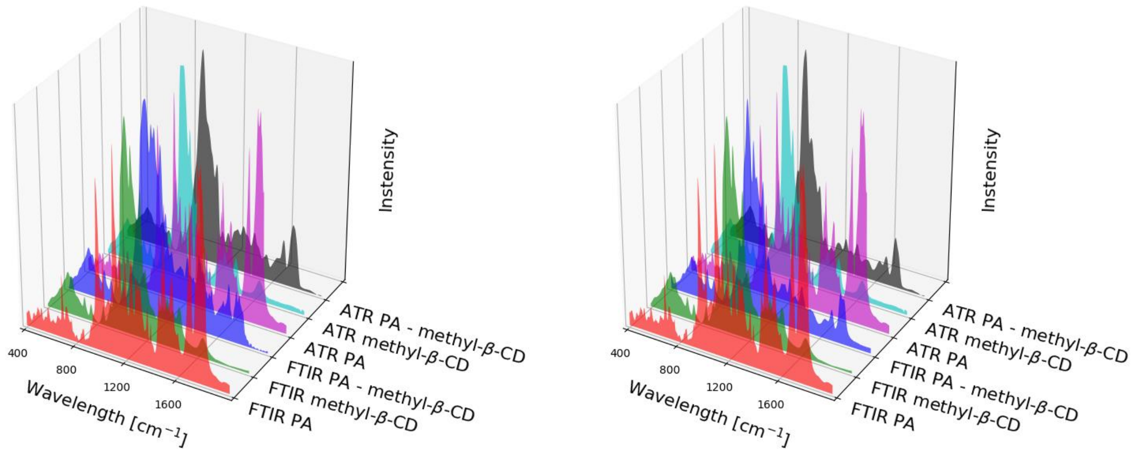

physical mixture *complex confirmed with DSC*

Figure S40. A records of input dataset containing features from FTIR and ATR spectra of PA – MβCD systems and its constituents.

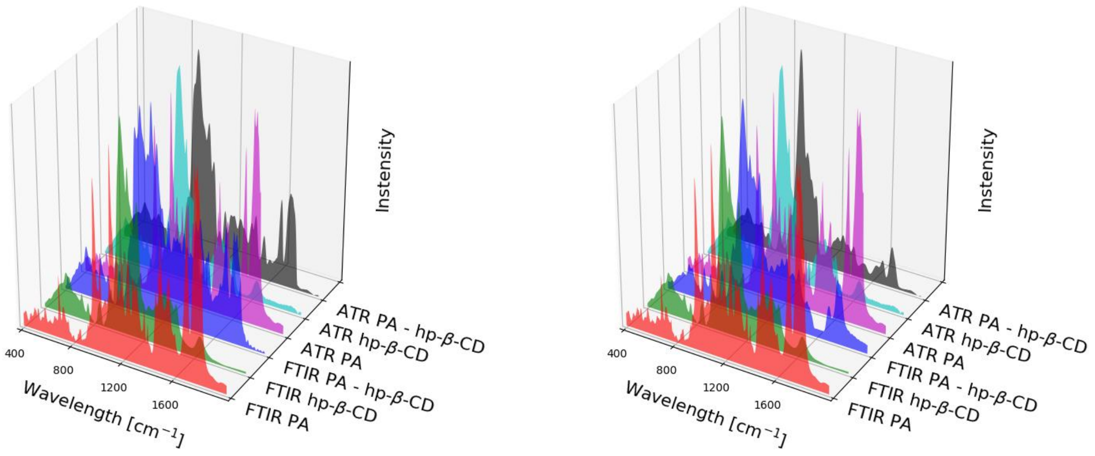

physical mixture *complex confirmed with DSC*

Figure S41. A records of input dataset containing features from FTIR and ATR spectra of PA – HPβCD systems and its constituents.

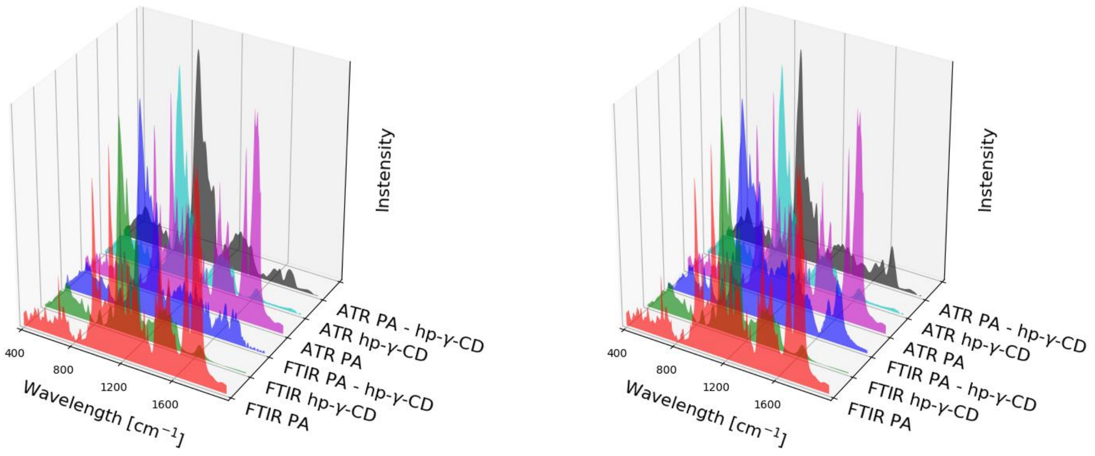

physical mixture *complex confirmed with DSC*

Figure S42. A records of input dataset containing features from FTIR and ATR spectra of PA – HPγCD systems and its constituents.

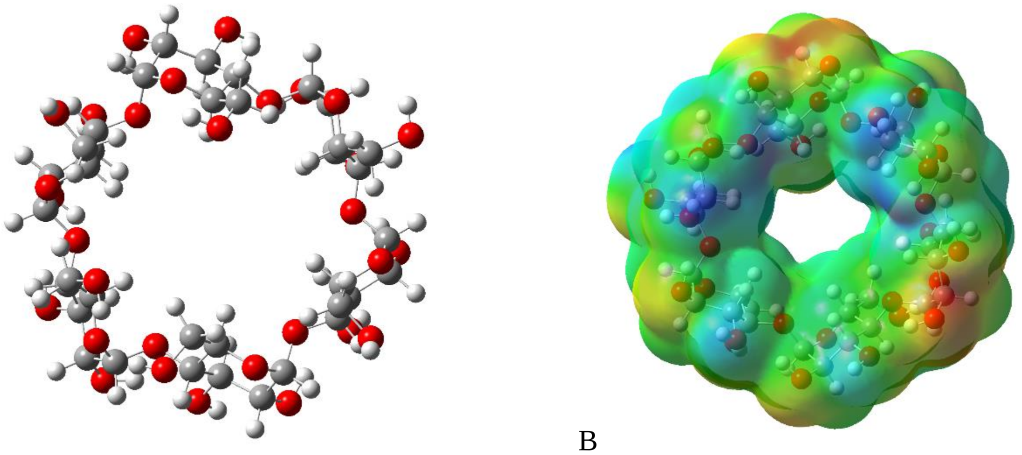

A B

Figure S43. Optimized structure of αCD (A) and its electrostatic potential map (B).

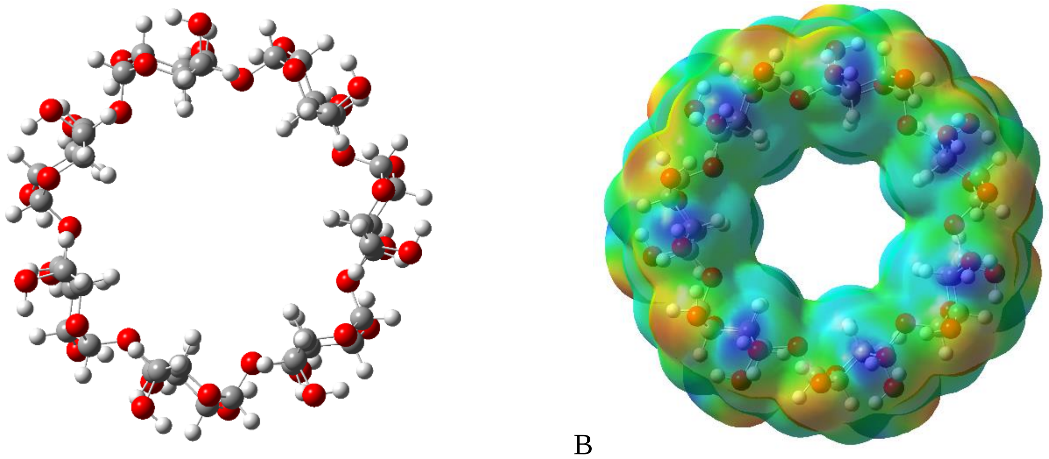

A B

Figure S44. Optimized structure of βCD (A) and its electrostatic potential map (B).

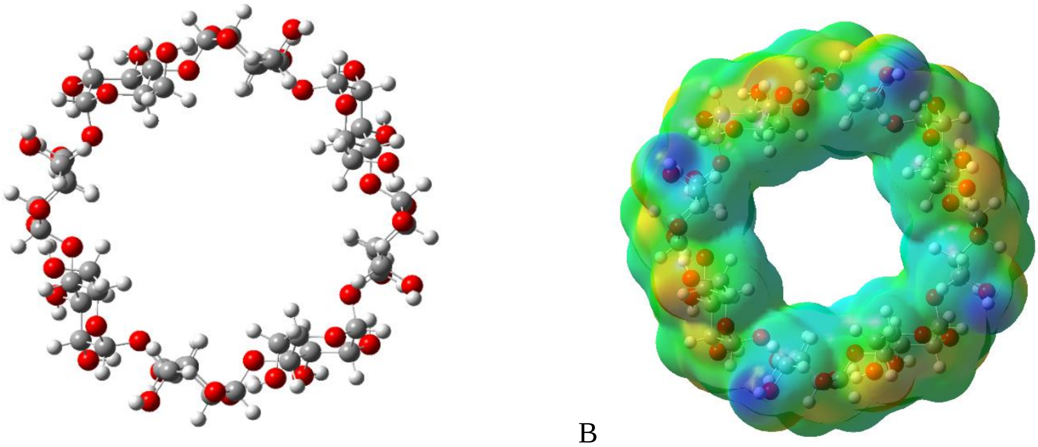

A B

Figure S45. Optimized structure of γCD (A) and its electrostatic potential map (B).

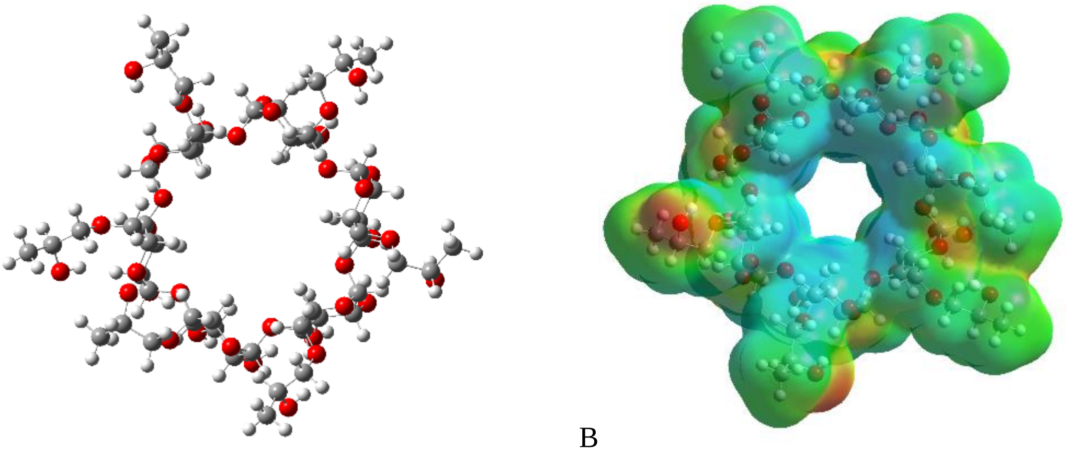

A B

Figure S46. Optimized structure of HPαCD (A) and its electrostatic potential map (B).

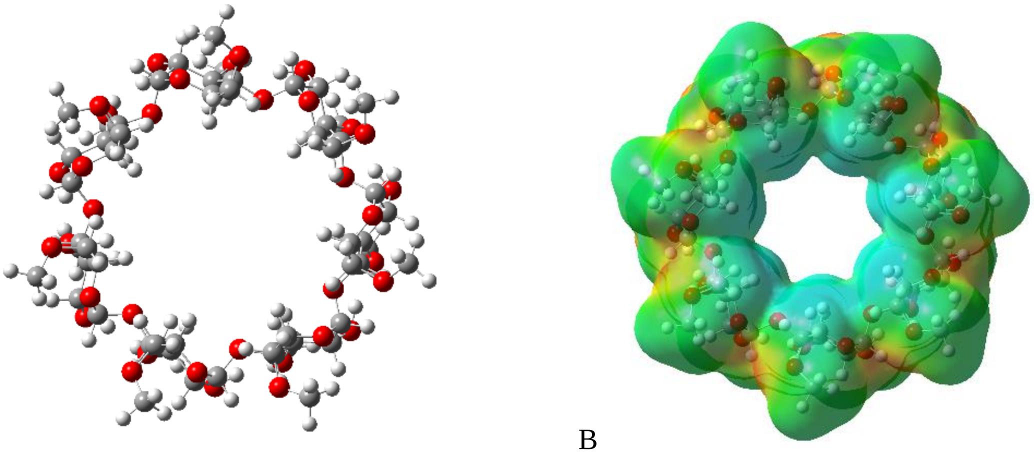

A B

Figure S47. Optimized structure of MβCD (A) and its electrostatic potential map (B).

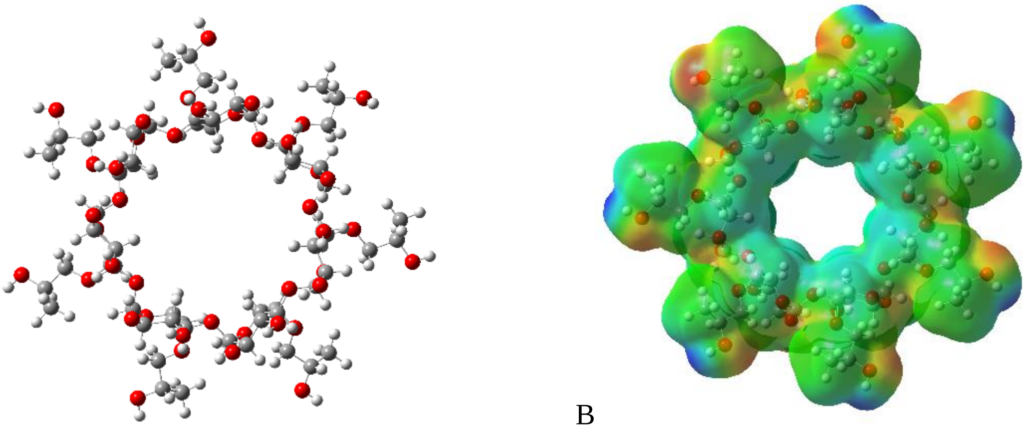

A B

Figure S48. Optimized structure of HPβCD (A) and its electrostatic potential map (B).

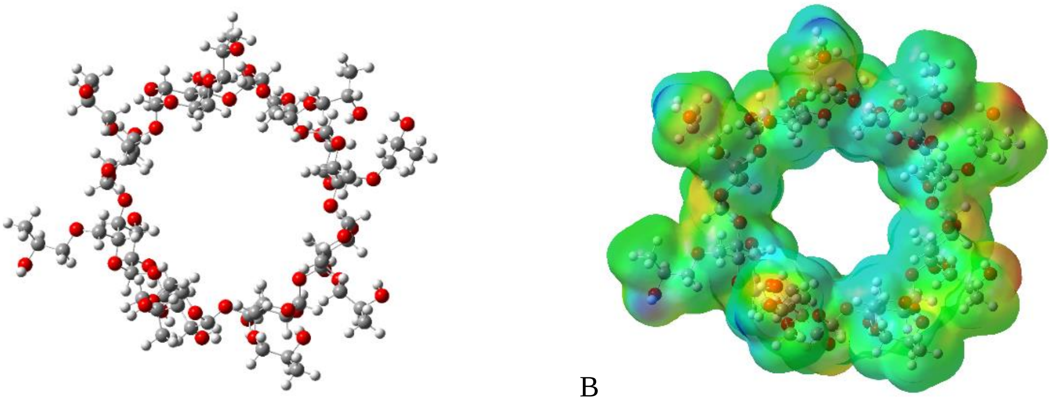

A B

Figure S49. Optimized structure of HPγCD (A) and its electrostatic potential map (B).

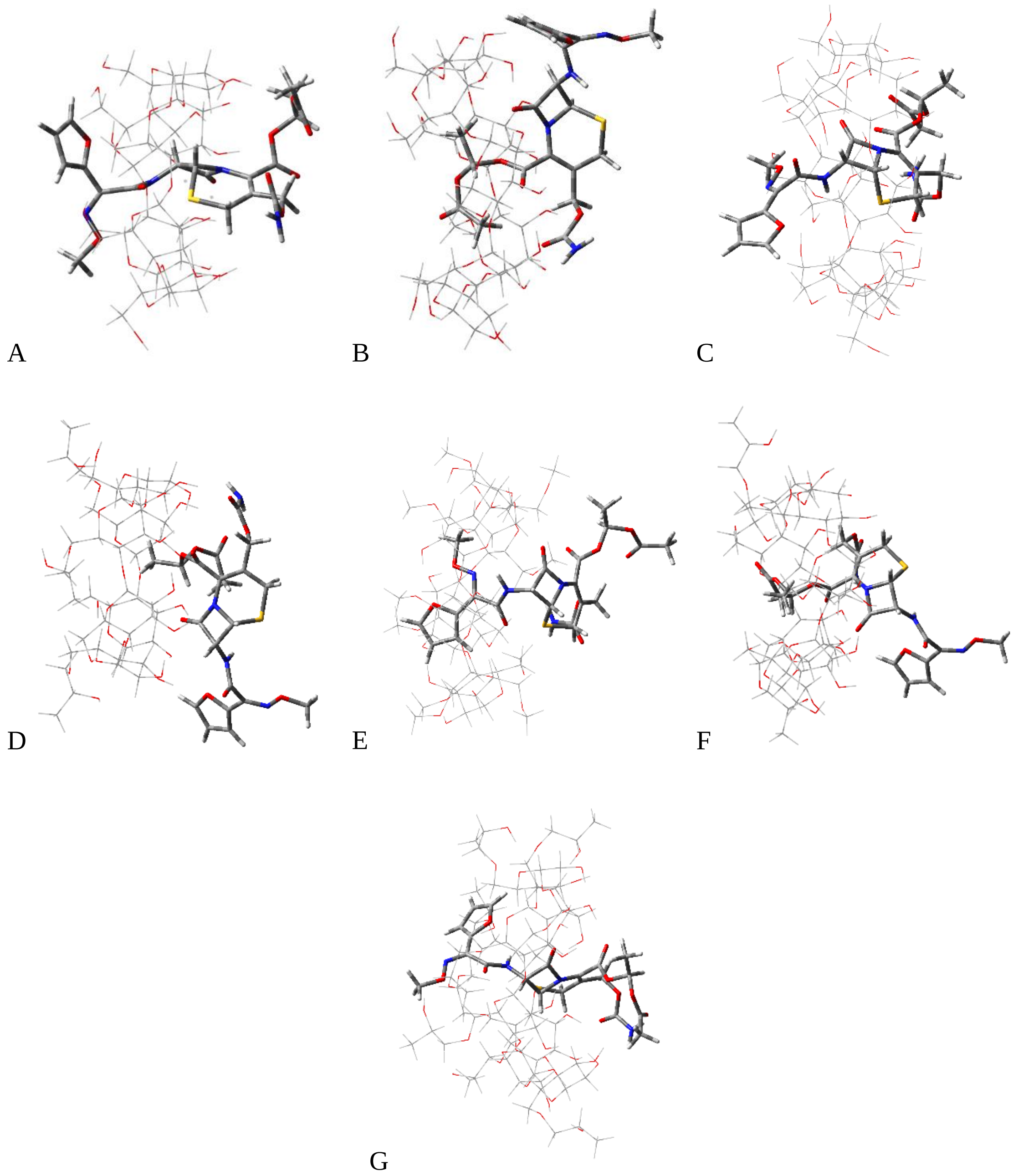

Figure S50. Binding modes of CA with αCD (A), βCD (B), γCD (C), HPαCD (D), MβCD (E), HPβCD (F), HPγCD (G).

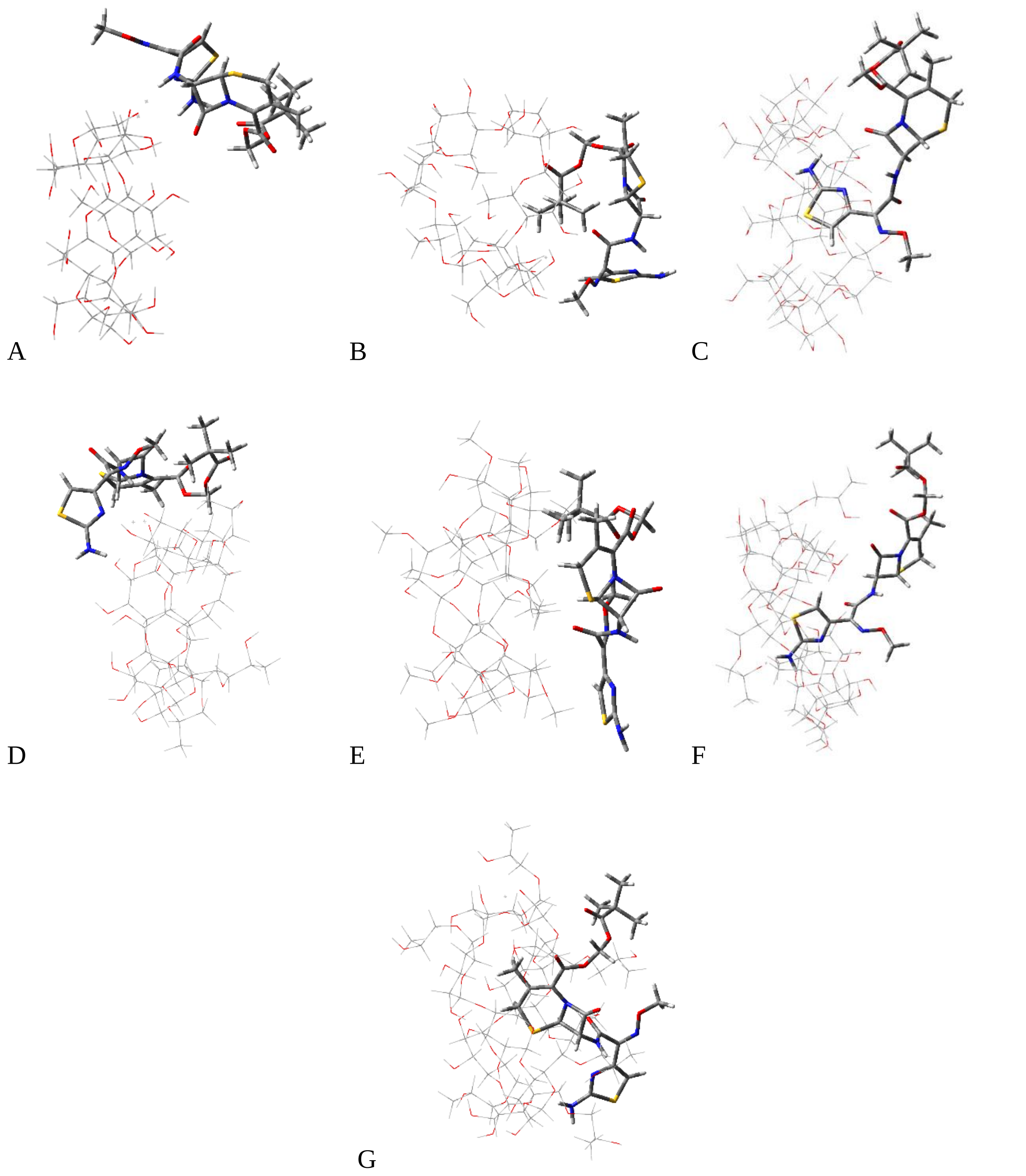

Figure S51. Binding modes of CT with αCD (A), βCD (B), γCD (C), HPαCD (D), MβCD (E), HPβCD (F), HPγCD (G).

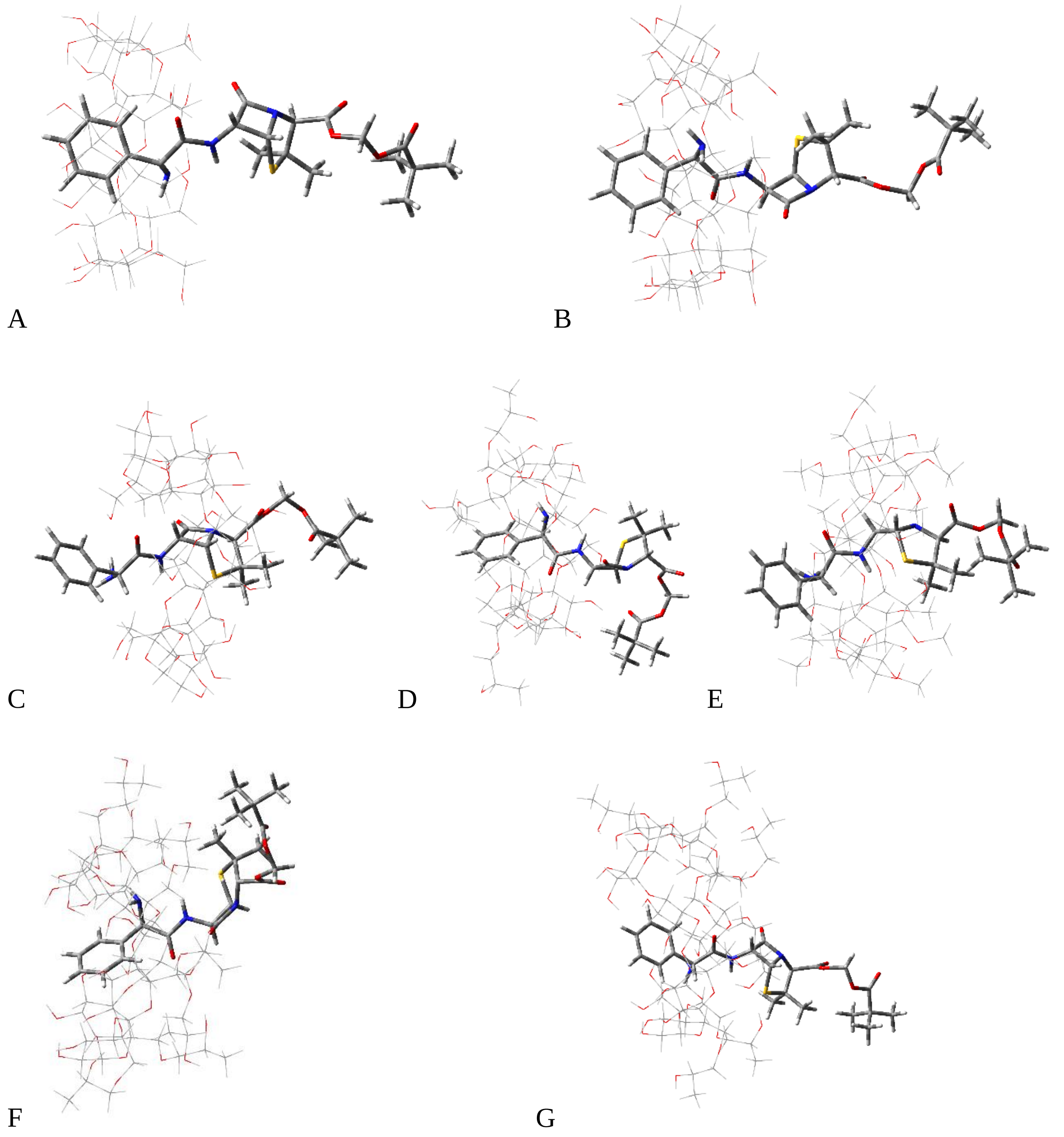

Figure S52. Binding modes of PA with αCD (A), βCD (B), γCD (C), HPαCD (D), MβCD (E), HPβCD (F), HPγCD (G).

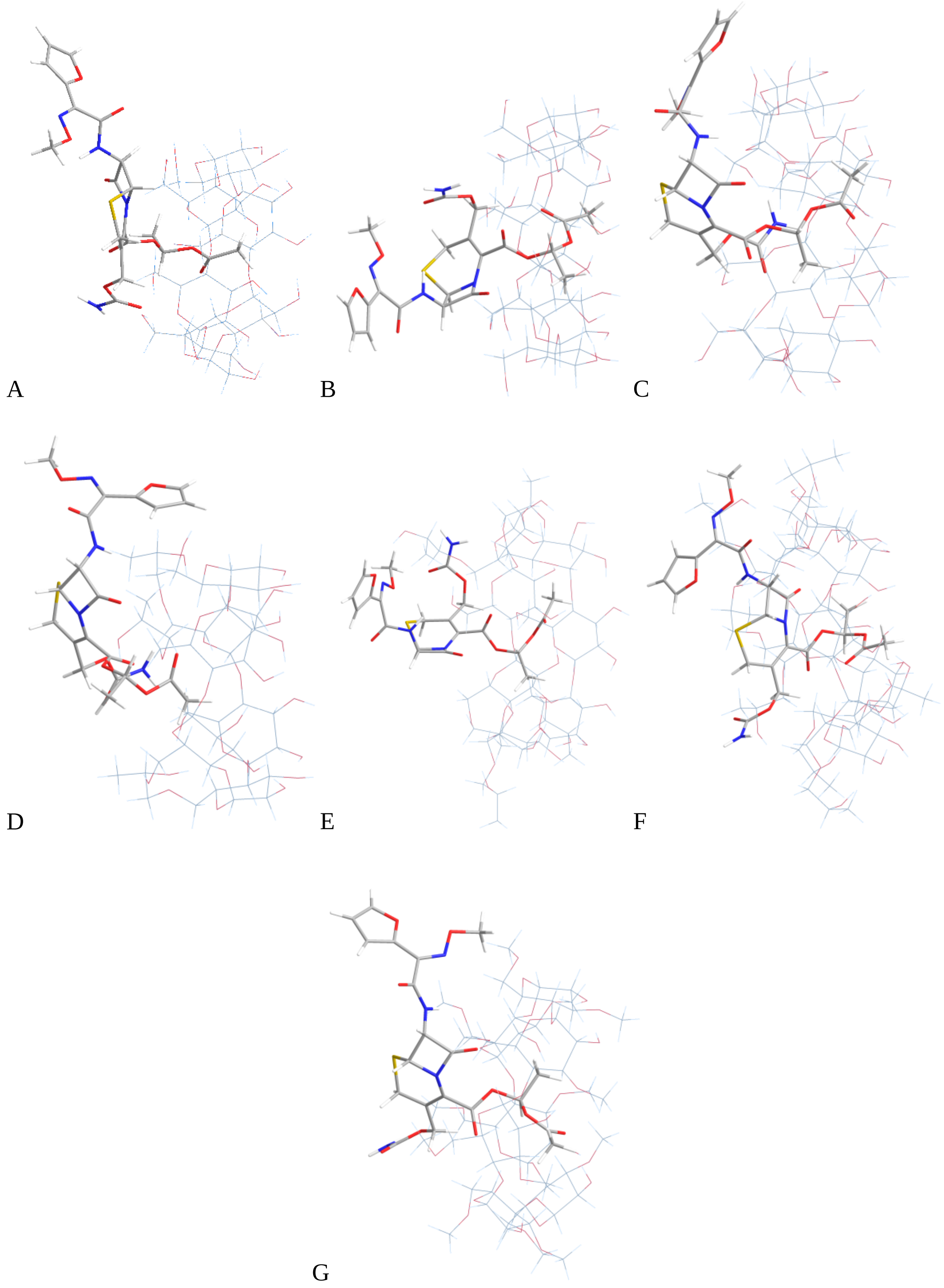

Figure S53. Binding modes of CA with αCD (A), βCD (B), γCD (C), HPαCD (D), MβCD (E), HPβCD (F), HPγCD (G) acquired according to machine learning study.

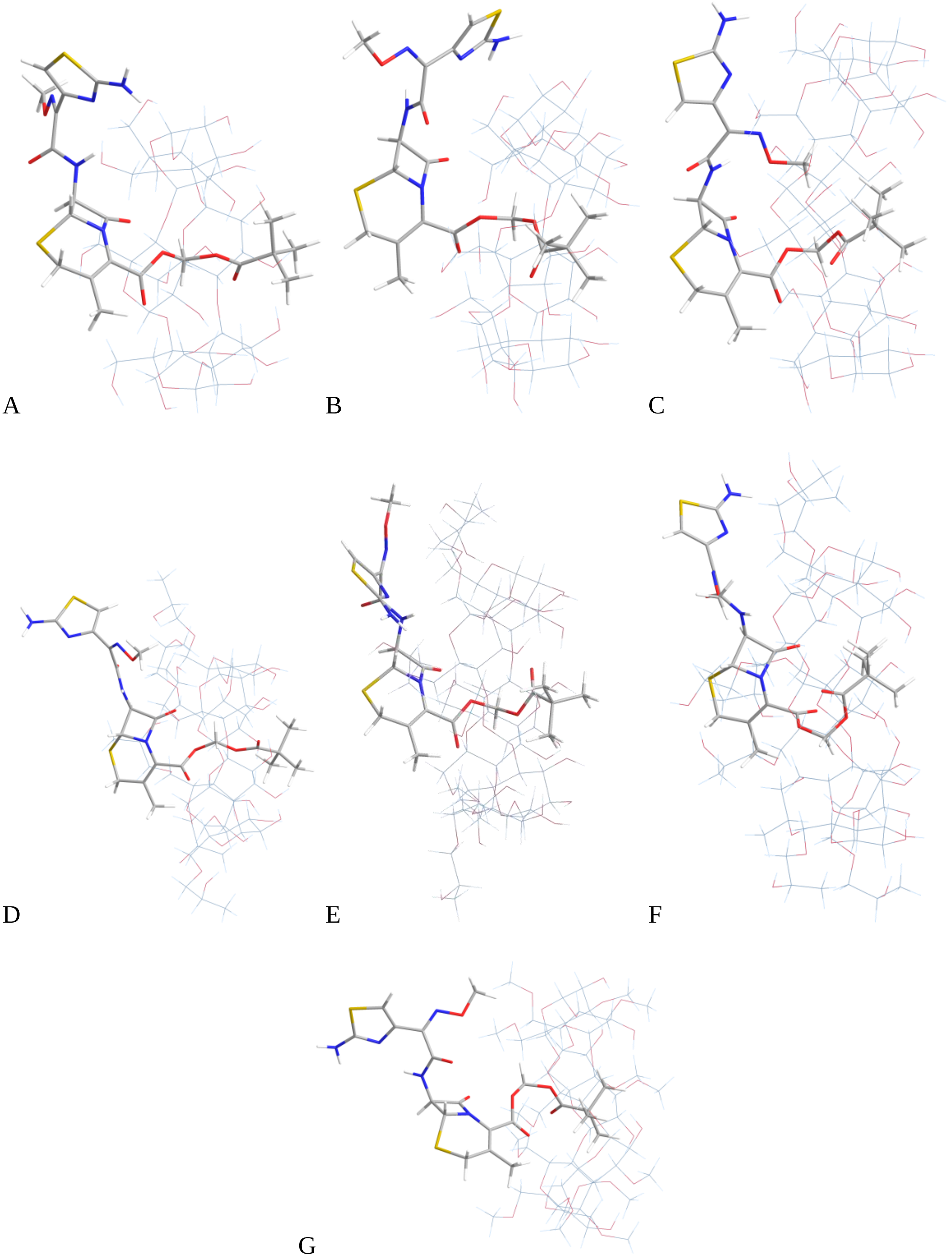

Figure S54. Binding modes of CT with αCD (A), βCD (B), γCD (C), HPα-CD (D), MβCD (E), HPβCD (F), HPγCD (G) acquired according to machine learning study.

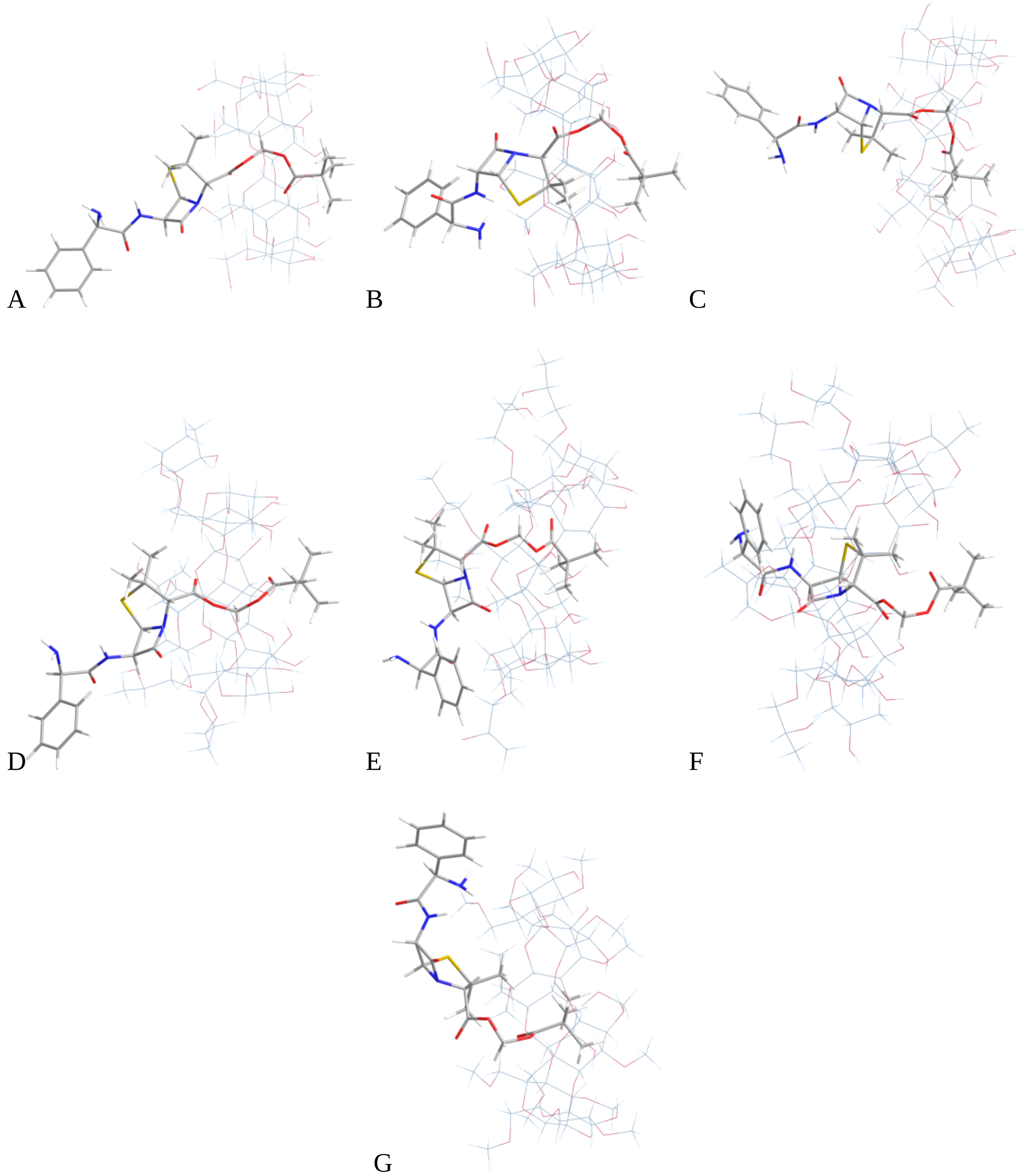

Figure S55. Binding modes of PA with αCD (A), βCD (B), γCD (C), HPαCD (D), MβCD (E), HPβCD (F), HPγCD (G) acquired according to machine learning study.
